# Developmental Changes in Genome Replication Progression in Pluripotent versus Differentiated Human Cells

**DOI:** 10.3390/genes15030305

**Published:** 2024-02-27

**Authors:** Sunil Kumar Pradhan, Teresa Lozoya, Paulina Prorok, Yue Yuan, Anne Lehmkuhl, Peng Zhang, M. Cristina Cardoso

**Affiliations:** 1 Cell Biology and Epigenetics, Department of Biology, Technical University of Darmstadt, 64287 Darmstadt, Germany; sunil_kumar.pradhan@tu-darmstadt.de (S.K.P.); prorok@bio.tu-darmstadt.de (P.P.);; 2 Center for Tissue Engineering and Stem Cell Research, Guizhou Medical University, Guiyang 550004, China; yuanyue5815@163.com

**Keywords:** human cells, induced pluripotent stem cells, pluripotent embryonic stem cells, genome replication progression, repli-FISH, rDNA, centromere, chromatin compaction

## Abstract

DNA replication is a fundamental process ensuring the maintenance of the genome each time cells divide. This is particularly relevant early in development when cells divide profusely, later giving rise to entire organs. Here, we analyze and compare the genome replication progression in human embryonic stem cells, induced pluripotent stem cells, and differentiated cells. Using single-cell microscopic approaches, we map the spatio-temporal genome replication as a function of chromatin marks/compaction level. Furthermore, we mapped the replication timing of subchromosomal tandem repeat regions and interspersed repeat sequence elements. Albeit the majority of these genomic repeats did not change their replication timing from pluripotent to differentiated cells, we found developmental changes in the replication timing of rDNA repeats. Comparing single-cell super-resolution microscopic data with data from genome-wide sequencing approaches showed comparable numbers of replicons and large overlap in origins numbers and genomic location among developmental states with a generally higher origin variability in pluripotent cells. Using ratiometric analysis of incorporated nucleotides normalized per replisome in single cells, we uncovered differences in fork speed throughout the S phase in pluripotent cells but not in somatic cells. Altogether, our data define similarities and differences on the replication program and characteristics in human cells at different developmental states.

## 1. Introduction

Spatio-temporal genome replication patterns correlate with the 3D genome organization and underlying chromatin structure [1,2]. Earlier studies of genome replication progression in mouse somatic cells using nucleotide analogs (5-bromo-2′-deoxyuridine (BrdU) and 5-ethynyl-2′-deoxyuridine (EdU)) coupled with sequencing analysis or fluorescence microscopy showed the correlation between DNA replication timing and increasing chromatin compaction [3,4]. In general, the open euchromatin replicates early, followed by facultative and constitutive heterochromatin replicating later in the S phase [5]. Developmental progress entails changes in 3D genome organization, and epigenetic marks, which eventually lead to differential gene expression. Such changes also affect the temporal replication program. However, increasing evidence suggests a role of genome replication programs and features underlying cell fate [6,7]. Hence, investigating developmental progression from the perspective of genome replication programs has become of great interest.

The intricate relationship between developmental change and replication timing is well-studied in mouse models [6,8]. Early in development, the genes involved in maintaining the pluripotency replicate earlier and change from early to late replicating upon differentiation. A similar observation was conducted for pericentromeric constitutive heterochromatin in mouse embryonic stem cells (mESC). Mouse ESC execute the genome replication differently, replicating constitutive heterochromatin earlier than facultative heterochromatin but upon differentiation the replication timing between constitutive and facultative heterochromatin switches, suggesting a role of repeat regions in cell fate and developmental regulation [6]. A similar pattern change has also been identified in Drosophila, where satellite sequences become progressively heterochromatic, and their replication is delayed upon differentiation at later developmental cycles [9]. In human diploid fibroblasts, during the first half of the S phase, subnuclear replication domains, known as replication foci (RFi), are located in the euchromatin regions; afterward, in the middle of the S phase, RFi are arranged at the nuclear periphery and in perinucleolar regions, and at the end of S phase, replication occurs at satellite heterochromatic regions [10].

Developmental processes and chromatin organization poised by epigenetic reprogramming influence the genome replication program. In *Drosophila*, *Xenopus*, and mice, several genome replication features such as fork speed, fork directionality, and origin firing underlie developmental changes [11]. Hence, it becomes crucial to dissect the global genome replication program and features underlying developmental processes. Efforts have been made in the past two decades to characterize the genome replication program underlying developmental changes in humans [12]. Such genome-wide studies provide genomic sequence context information but lack 3D spatial information as well as have a low temporal resolution. Importantly, there is little information regarding repeat elements, which constitute more than half of the human genome.

Here, we analyzed the developmental differences in the replication progression pattern in human iPSC, human ESC, and human somatic cells. We characterized the global spatio-temporal replication pattern, together with replicon quantification, comparing the results among the different human cell lines. We found that the spatial organization of RFi at the late stage of the S phase in somatic cells is distinct from the one of pluripotent cells. A fork efficiency measurement and genome-wide origin mapping were performed and demonstrated that the number of replication origins decreased during cell differentiation. Furthermore, chromatin compaction classification analysis in combination with histone modification analysis reveals differences in chromatin dynamics. Finally, by analyzing in detail the replication timing of genomic repeat elements, various differences among differentiated and pluripotent cells could be measured, especially in the replication of rDNA repeats, which showed a delayed replication timing in human pluripotent cells.

## 2. Materials and Methods

### 2.1. Cell Culture and Transfection

The details of the cell lines used are described in Table 1. All cells were grown in a humidified atmosphere of 5% CO_2_ at 37 °C. Human hTERT RPE1 and BJ-5ta cells were grown in Dulbecco’s Modified Eagle Medium (DMEM) (Cat.No.: D6429, Sigma-Aldrich Chemie GmbH, Steinheim, Germany) supplemented with 10% fetal calf serum (Cat.No.: FBS 11A, Capricorn Scientific GmbH, Hessen, Germany), 1x glutamine (Cat.No.: G7513, Sigma-Aldrich, St Louis, MO, USA), 1 µM gentamicin (Cat.No.: G1397, Sigma-Aldrich, St Louis, MO, USA), and 0.01 mg/mL hygromycin B (Cat.No.: 843555, Roche, Basel, Switzerland). To grow the hESC H1, hiPSC A4, and hiPSC B4, surfaces were first coated with vitronectin (Cat.No.: A14700, ThermoFisher Scientific, Waltham, MA, USA) for one hour. The hiPSC A4 and hiPSC B4 were grown in iPSC Brew Basal medium (Cat.No.: 130-107-086, Miltenyi Biotec, Teterow, Germany) supplemented with iPSC-Brew 50x (Cat.No.: 130-107-087, Miltenyi Biotec, Bergisch Gladbach, Germany). The hESC H1 was grown in mTeSR™1 (Cat.No.: 85850, STEMCELL Technologies, Cambridge, MA, USA) on Matrigel (Cat.No.:354277, Corning, NY, USA)-coated plates. All hESC and hiPS cells were grown till they started forming colonies before performing experiments.

For live-cell time-lapse microscopy, cells were transfected with the plasmid pENeGFPCNAL2mut (pc0653, https://www.addgene.org/167564/ accessed on 25 January 2024) [4]. The hTERT RPE1 cells were transfected with the AMAXA Nucleofector II system (Lonza, Cologne, Germany), using a self-made buffer (5 mM KCl, 15 mM MgCl_2_, 120 mM Na_2_HPO_4_/NaH_2_PO_4_ pH 7.2, 50 mM Mannitol) with the program A024 and seeded on a polymer coverslip bottom µ-slide 8-well plate (Cat.No.: 80826, Ibidi, WI, USA). The hiPSC A4 cells were first seeded till they form colonies on vitronectin coated polymer coverslip bottom µ-slide 8-well plate transfected with Lipofectamine™ Stem Transfection Reagent (Cat.No.: L300015, ThermoFisher Scientific, Waltham, MA, USA) using the manufacturer’s recommended protocol.

### 2.2. Doubling Time and (sub)S Phase Duration

For doubling time/cell cycle length quantification, two-time points falling within the logarithmic phase of cell proliferation (cell confluency between 30 and 70%) were used. First, 1 × 10^5^ hTERT RPE1, hiPSC A4, hiPSC B4, and hESC H1 cells were seeded as technical triplicates. The counting started once the cells became adherent to and started forming colonies. Cells were trypsinized and resuspended in 1X PBS. Cell numbers were counted with a Neubauer hemocytometer for multiple time points within a 24 h interval. Doubling time (*dt*) of the cell culture was then calculated by dt=(log2×Δt)÷(logN2−logN1), where *N*1 and *N*2 are the numbers of cells counted at time point 1 and 2, respectively, and ∆*t* is the duration between the two time points.

To determine the percentage of cells in the S phase, asynchronously growing cell populations were pulse-labeled with 10 µM of nucleoside analog 5-ethynyl-2′-deoxyuridine (EdU) (Cat.No.: 7845.1, ClickIT-EdU cell proliferation assay, Carl Roth, Karlsruhe, Germany) for 15 min, formaldehyde fixed, and EdU was detected along with 4′,6-diamidino-2-phenylindole (DAPI) as described below. High-throughput images were acquired and analyzed as described below. Based on EdU and DAPI intensity, the cell cycle profile was plotted, and the fraction of cells in each cell cycle was determined. To determine the duration of the S phase, the fraction of cells in the S phase was multiplied by cell cycle duration. To determine the percentage of cells in each S phase, the number of cells in each S phase was manually counted by scoring the EdU spatial patterns from images acquired using high throughput microscopy using a 40x objective. The fraction of cells in each S phase was multiplied by doubling time duration to calculate the duration of each S phase stage.

### 2.3. Genome Replication Labeling and Visualization

A list of all the nucleotide/nucleoside analogs and antibodies used is described in Table 2 and Table 3, respectively.

*Pulse labeling:* For the replication labeling and visualization experiments, the cells were seeded on sterilized coverslips with respective media. The cells were pulse-labeled with 10 µM of EdU for 15 min before washing with PBS 1× and fixing with 3.7% formaldehyde in PBS 1× for 10 min.

*Pulse-chase–pulse-chase:* Cells were seeded on sterilized coverslips with respective media. First, cells were incubated with 10 µM of EdU for 15 min (first pulse). The cells were washed twice with respective warm media supplemented with 50 µM of thymidine (Cat.No.: T1895, Sigma-Aldrich Chemie GmbH, Taufkirchen, Germany) to stop the incorporation of EdU before incubating with fresh media for another three hours. Cells were then incubated with 10 µM of 5-bromo-2′-deoxyuridine (BrdU) (Cat.No.: B5002, Sigma-Aldrich Chemie GmbH, Taufkirchen, Germany) for 15 min (second pulse). The cells were washed twice with warm media supplemented with 50 µM of thymidine and incubated in fresh media for another three hours. The cells were washed with PBS 1× before fixing with 3.7% formaldehyde in PBS 1× at room temperature for 10 min. After fixation, the cells were washed thrice with PBS 1×.

*Immunofluorescence staining:* All the immunostaining was performed inside a dark, humidified chamber at room temperature unless otherwise mentioned. After fixation, the cells were permeabilized with 0.5% Triton X-100 (Carl Roth, Karlsruhe, Germany), in PBS 1× for 10 min, followed by three washes with 0.05% Tween in PBS 1×. To give access to the PCNA epitope, the cells were incubated with ice-cold methanol for 10 min. The cells were again washed thrice with a washing buffer (0.05% Tween in PBS 1×) and blocked with 4% BSA in PBS 1× for 30 min.

For the detection of EdU, cells were incubated in Click-IT cocktail mix 100 mM Tris-HCl pH 8.5, 10 mM CuSO_4_, 1 µM 647 Azide (Cat.No.: 259P.1, Carl Roth, Karlsruhe, Germany), and 100 mM ascorbic acid diluted in water for 30 min [16]. Cells were washed thrice with 0.05% Tween in PBS 1×.

To detect BrdU, cells were incubated in anti-BrdU primary antibody diluted in 2% BSA, 1× DNase I buffer (60 mM Tris/HCl pH 8.1, 0.66 mM MgCl_2_, 1 mM β-mercaptoethanol), and 0.1 U/mL DNase I (Cat.No.: D5025, Sigma-Aldrich Chemie GmbH, Steinheim, Germany) for one hour at 37 °C. For the inactivation of DNase I, cells were washed twice with EDTA PBS 1× for 10 min each.

For PCNA detection, cells were incubated in the primary antibody for two hours, and washed thrice with 0.05% Tween in PBS 1× before adding suitable secondary antibodies for one hour and washing.

To detect the histone modifications, cells were blocked in the blocking buffer (4% BSA/1% fish skin gelatin/PBS 1×) and incubated in the respective primary antibodies overnight at 4 °C. The cells were washed five times and incubated in suitable secondary antibodies for one hour before washing five times with washing buffer.

The cells were stained with 10 mg/mL DAPI (4′,6-diamidino-2-phenylindole, Cat.No.: D9542, Sigma-Aldrich Chemie GmbH, Steinheim, Germany) for 10 min and mounted on Vectashield (Cat.No.: VEC-H-1000, Vector Laboratories Inc., Burlingame, CA, USA). All the coverslips were sealed with transparent nail polish and air dried.

### 2.4. Probe Generation, Metaphase Spread, Repli-FISH, and Immuno Repli-FISH

*Probe generation:* The probe generation, fluorescence in situ hybridization, and co-detection of replication foci (RFi) and FISH probes experiments were performed as described before [17]. All the plasmids and primers used are summarized in Table 4. For the genomic DNA (gDNA) preparation, hTERT RPE1 was pelleted and incubated overnight in TNES buffer (10 mM Tris; pH 7.5, 400 mM NaCl, 10 mM EDTA, 0.6% SDS) supplemented with 1 mg/mL Proteinase K (Cat. No.:BS202505, Bio&sell GmbH, Feucht, Germany) at 50 °C. RNA was removed by the addition of 0.5 mg/mL RNase A (Cat.No.: 10109169001, Sigma-Aldrich Chemie GmbH, Steinheim, Germany) for 30 min at 37 °C. The gDNA was extracted by the addition of 6 M NaCl at a final concentration of 1.25 M and vigorous shaking. After centrifugation (15 min, 11,000× *g*, RT), gDNA was precipitated from the supernatant by the addition of 100% ice-cold ethanol followed by incubation at –20 °C for 1 h and subsequent centrifugation (10 min, 11,000× *g*, 4 °C). The pellet was washed with 70% ethanol, air dried, and dissolved in double distilled water. The plasmids containing rDNA and LINE1 probes were labeled with Cy3-dUTP (Cat.No.: ENZ-42501, Enzo Life Sciences, Lörrach, Germany) using nick translation. To prepare the Alu and centromere probes, the purified gDNA from hTERT RPE1 was used as a template to amplify and label with biotin-16-dUTP (Cat.No.: 11093070910, Roche Diagnostics Deutschland GmbH, Mannheim, Germany) via PCR using specific Alu primers (5′-GGATTACAGGYRTGAGCCA-3′; 3′-RCCAYTGCACTCCAGCCTG-5′) as well as specific centromere primers (α27: 5′-CATCACAAAGAAGTTTCTGAGAATGCTTC-3′); (α30: 5′-TGCATTCAACTCACAGAGTTGAACCTTCC-3′). Optionally, probes were sheared with a Covaris S220 (Covaris Inc., Woburn, MA, USA) in microTUBEs (50 µL aliquots; 520,045, Covaris Inc.) to a final size of ~ 500 bp when the size distribution of the labeled probes was above 2 kb. All probes (~100 ng) except rDNA were precipitated with 1 µg of fish sperm DNA (Cat.No.: 10223638103, Roche Diagnostics Deutschland GmbH, Mannheim, Germany), 0.13× NaAC, and 2.5× ethanol, before being washed with 70% ethanol, air dried, and dissolved in the hybridization solution (50% Formamide/SSC 2×). Around 100 ng of rDNA was co-precipitated with human 1 µg of Cot-1 DNA (Cat.No.: 5190-3393, Agilent, Santa Clara, CA, USA), 1 µg of fish sperm DNA, 0.13× NaAC, and 2.5× ethanol to reduce non-specific signals.

*Metaphase spreads* were used to validate the probes. The hTERT RPE1 cells were seeded for at least 24 h before being treated with 0.1 µg/mL colcemid (N-deacetyl-N-methylcolchicine, Cat.No.: 10295892001, Roche Diagnostics Deutschland GmbH, Mannheim, Germany) for three to four hours. Cells were then harvested by trypsinization and incubated for 30 min with 75 mM KCl at 37 °C with tapping in between. They were then fixed dropwise by adding ice-cold methanol/acetic acid (3:1) for 30 min on ice and this was repeated twice. For chromosome spread, the cell suspension was dropped onto an ice-cold wet microscopy slide from a height of approximately 20 cm. The slide was then air dried overnight. For metaphase FISH, the slides were rehydrated in ddH_2_O for 10 min, digested with 0.005% pepsin (165 U/mL, Cat.No.: P6887, Sigma-Aldrich Chemie GmbH, Steinheim, Germany) in 0.01 M HCl for 10 min at 37 °C, washed twice with SSC 2×, dehydrated in 70 and 100% ethanol for 3 min each and air dried. After equilibrating with 5 µL of hybridization solution containing respective probes for 30 min at 37 °C inside a sealed hybridization box, the metaphase spreads were co-denatured at 80 °C for five minutes and immediately placed on ice for another five minutes. The box was then transferred to a humidified chamber (37 °C) and left overnight. Post-hybridization washes were performed with SSC 2× and blocked with 2% BSA/SSC 2× for 30 min. Biotin-labeled probes were detected with a suitable streptavidin-conjugated fluorophore (see Table 3), counterstained with DAPI, and mounted on Vectashield.

*Repli-FISH and immuno-FISH*: Cells were treated with 10 µM of EdU for 15 min, washed twice with PBS 1×, and fixed with 3.7% formaldehyde in PBS 1×. The cells were permeabilized with 0.5% Triton X-100 in PBS 1×, washed, and incubated in 20% glycerol in PBS 1× overnight at 4 °C. The cells were snap-frozen in liquid nitrogen and washed with PBS 1×, and this step was repeated two more times. The detection of EdU was performed before the FISH as above and fixed with 2% formaldehyde in PBS 1× for 10 min. The DNA was depurinated in ice-cold 0.1 N HCl/0.5% Triton X-100 for 5 min, the cells were washed with SSC 2×, before incubating with the probes. For Alu and LINE1, equal volumes from each probe were pulled, mixed, and added to the coverslip, incubated for 15 min at 37 °C. In a water bath, the cells and probes were co-denatured at 80 °C for five minutes, immediately placed on ice for five minutes, transferred to the humidified hybridization chamber at 37 °C, and left overnight. The cells were washed thrice in the washing buffer (0.05% Tween in SSC 4×) and blocked in the blocking buffer. The probes were detected with a streptavidin conjugated fluorophore. A sequential hybridization was performed for co-detection of centromere and rDNA as the rDNA probe contained the Cot-1 DNA. Cells were treated with RNAse I before proceeding with the centromere hybridization, washing, and detection. It was followed by rDNA hybridization and post-hybridization washing. The detection of PCNA was performed after the FISH as described above.

Before detecting RPA 194, EdU was detected and fixed with 2% formaldehyde for 10 min, and rDNA hybridization was performed. After washing and blocking (with 4% BSA/1% fish skin gelatin/PBS 1x), the primary antibody for RPA 194 was added for two hours, washed five times with 0.05% Tween in PBS 1×, and incubated in secondary antibody for one hour. The cells were further washed five times with 0.05% Tween in PBS 1×.

All cells were fixed with 1% formaldehyde/PBS 1x for three minutes, washed twice with PBS 1x, counterstained with DAPI, and mounted on Vectashield. The coverslips were sealed with transparent nail polish and air dried.

### 2.5. Microscopy

A list of microscopic systems used is available in Table 5.

High throughput image acquisition was performed on a Nikon CREST system with a 20× objective.

Live-cell imaging was performed on a Nikon CREST microscope (Nikon GmbH) system with a 40× objective. Time-lapse microscopy was conducted in a closed live-cell chamber at 37 °C, with 5% CO_2_ and 60% humidity. The z-stacks of the transfected cells were taken every 45 min for 24 h. The media was supplemented with 50 µM of Trolox ((±)-6-hydroxy-2,5,7,8-tetramethylchromane-2-carboxylic acid, Cat.No.:238813, Sigma-Aldrich Chemie GmbH, Taufkirchen, Germany).

The pulse-chase–pulse-chase 3D image acquisitions were performed on a Leica SP5 II confocal microscope. The pulse, immunostaining, repli-FISH, and immuno-FISH images were acquired using a Zeiss Airyscan 2 microscope in 3D. Raw images were taken in airyscan mode and further processed with airyscan joint deconvolution to generate super-resolved images on Zen software.

### 2.6. Image Analysis

A list of image analysis software and associated plugins/packages are listed in Table 6. A folder containing all the scripts used is uploaded here (https://tudatalib.ulb.tu-darmstadt.de/handle/tudatalib/4110.2 (accessed on 25 January 2024)). All image analysis was performed either on FiJi or R. All 16-bit images were first converted to 8-bit using script “ASJD_16_to_8_bit.ijm” before analysis unless otherwise mentioned.

**Figure 1:**
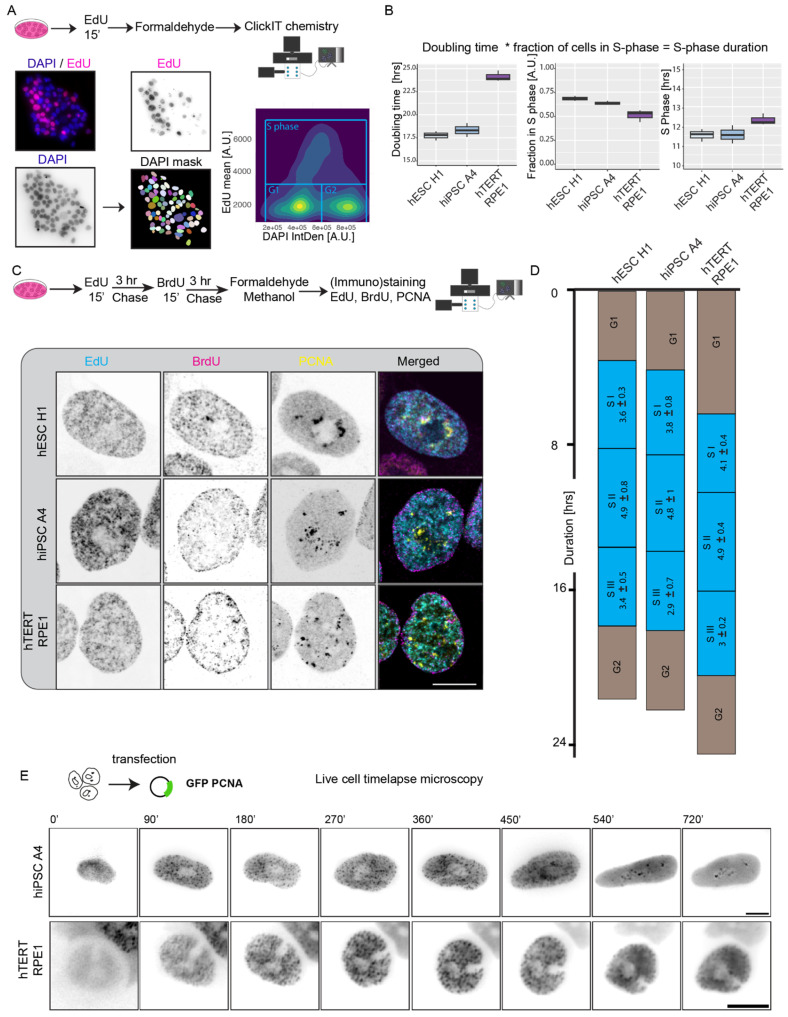
Cell cycle and replication dynamics analysis of pluripotent and somatic cells. (**A**) Schematic representation of how the fraction of cells in S phase was determined based on DAPI and EdU intensity using high-throughput microscopy. (**B**) Doubling time of hESC H1, hiPSC A4, and hTERT RPE1 was calculated by counting cell numbers at different time points from a defined number of seeded cells. The S phase fraction was calculated by dividing the EdU positive cells with the total number of cells from high-throughput image analysis. The S phase duration was calculated by multiplying the doubling time with the fraction of cells in the S phase. (* = multiplication) (**C**) A pulse-chase–pulse-chase experiment followed by replication foci (RFi) detection at three time points in the same cell in different cell lines. (**D**) Illustration shows cell cycle phases and (sub)S phase durations among cell types. The duration of each S phase sub-stage was calculated by multiplying the fraction of cells in each sub-stage by the doubling time of the specific cell. (**E**) Live-cell time-lapse microscopy of hiPSC A4 and hTERT RPE1 expressing GFP-PCNA showing genome replication progression. PCNA S phase foci are visible from 90 min on. For more details see Table S1. Scale bar: 10 µm.

*High-throughput image analysis:* The 3D images of the images were processed using process > z-project (sum slices) to obtain multichannel 2D images on FiJi. The StarDist plugin was used to segment the nuclei and masks were saved. The nuclear masks were used to measure both DAPI, and EdU intensity using ROI manager on FiJi (Figure 1A).

*High-resolution image analysis*: Prior to all image analysis, 3D nuclei were segmented using DAPI signal to define the nuclear region of interest (ROI) (Figure S1A). First, the 3D stack of DAPI was processed with a Gaussian filter (pixel radius = 2) and normalized (process > enhance contrast > saturated pixels = 0). The nucleus was segmented with 3D nuclei segmentation in the 3D suite plugin, a binary image was created, and further processed with dilations, fill holes, and erode (see script “Batch_DAPI_Segmentation_Process_3D.ijm”). This ROI was applied to all the channels to define the ROI using image calculator applying “min” between the DAPI mask and the channel of interest (see script “Image_Calculator_Min_Stack.ijm”). All DAPI masks were manually cross-checked as some of the hiPS cells can form a cylindrical shape, leading to suboptimal DAPI segmentation.

*RFi/replicon/FISH (3D spot) quantification:* A combined approach of threshold and cluster-based segmentation was performed for the 3D spot segmentation. First, the 3D stack was processed using a mean filter (pixel radius = 1) and normalized. The FindStackMaxima Macro (https://imagej.nih.gov/ij/macros/FindStackMaxima.txt) was used to find all the local maxima. The corresponding voxels of 3D Maxima were extracted from the processed image using image calculator, which was used as a seed (“Image_Calculator_Min_Stack.ijm”). The seed and processed images were used to segment the RFi/replicons/FISH spots in 3D spot segmentation (3D suite) using a Gaussian fit to determine the intensity value used as the threshold to stop the voxel clustering. The segmented objects were further processed with a 3D watershed split (3D suite) to separate the closely clustered RFi/replicons/spots. This was imported to 3D ROIManager (3D suite) for further quantifications and measurements (Figure S1B).

For 3D spot quantification, the 3D numbering plugin was used (RFI_or_Object_Quantification_in_ROI_DAPI_MASK.ijm) where the number of 3D spots was measured inside a single nucleus. The RFi distance analysis and features were extracted using the 3D ROIManager, where the first object was DAPI mask, and rest of the objects were individual RFi. All possible combinations of distances were measured using “distances” function (Figure S1B). The volume of individual RFi was measured using Measure 3D function. The segmented RFi was also overlaid on other channels (FISH, histone modifications) to measure the intensity distribution at the RFi (see script “Batch_Measurement_3DROIManager”).

*Repli-FISH using “AND” logic*: The FISH signals were processed using a mean filter (pixel radius = 1) and normalized. This was then segmented with simple segmentation (3D suite) to generate 3D ROIs and was imported to 3D ROIManager (see scripts “Repli_FISH”). A novel approach was used to directly measure the replication timing more accurately by using binary masks of the RFi and FISH, processing these masks using image calculator with “AND” logic operation to segment the repeat regions having the RFi. These ROIs were imported to 3D ROImanager for quantification.

*Chromatin compaction analysis*: Only 16-bit super-resolved images were analyzed using “Nucim” library available on platform “R” to subdivide individual nuclei into chromatin compaction classes and mapping signals from other channels to individual compaction classes. First, the DAPI channel was segmented and used to mask the region of interest. Individual voxels within a single nucleus were assigned to a certain compaction class based on the probability of this voxel belonging to the same class computed from a hidden Markov random field (HMRF) stochastic model, classifying the nuclei into seven different compaction classes, where the first class represents interchromatin (IC), two, three, and four represent active nuclear compartment (ANC), and last three classes represent chromatin domain clusters (CDC) (Figure S2A). Spots from other channels were further mapped into these subclasses based on a combined threshold and intensity method where first the spots were segmented with the threshold method followed by an intensity-weighted calculation of the relative fraction, leading to more intense signals having a larger impact and low-intensity signals having less impact (Figure S2B).

### 2.7. Genome-Wide Origin Mapping

The SNS-seq datasets (Table 7) were downloaded from the GEO database (Gene Expression Omnibus, https://www.ncbi.nlm.nih.gov/geo/ (accessed on 25 January 2024)) using sratoolkit (version 2.11.0). The reads were trimmed using Trimmomatic (version 0.36) and the quality of reads was evaluated using FastQC (version 0.11.9). Subsequently, the trimmed reads were aligned to the human genome (hg38 genome assembly, https://hgdownload.soe.ucsc.edu/goldenPath/hg38/bigZips/ (accessed on 25 January 2024)) using bowtie2 (version 1.3.1), (parameters: --very-sensitive, --end-to-end). Samtools (version 1.10) was used to generate bam files. The position of replication origins was determined using macs2 (version 2.1.2) (as narrow peaks, command: macs2 callpeak -t sample --gsize 2.7 × 10^9^ -q 0.001 --min-length 200 without control as no gDNA/RNAse control sample was available for those experiments). For the GSE128477 dataset, the peak references downloaded from the GEO database were used. The hESC-specific origins identified in GSE128477 and GSM927236 were concatenated and merged within a 1 kb window. The origin clustering was performed using bedtools (version bedtools/2.29.2), command bedtools merge at specified distances. The inter-origin distances (IOD) were calculated as distances between the mid positions of peaks. For visualization purposes, bam files corresponding to different experiments replicates were merged (command samtools merge) and converted to normalized bigWig files using deepTools (version 3.5.1_singularity, command: bamCoverage --bam smergedBam --normalizeUsing CPM --binSize 10). IGV (version 2.16.1) was used for visualization of representative SNS-seq signals. Other graphical representations (barplots, box plots, venn diagram) were realized in RStudio (Version 2023.03.1+446) using ggplot2 and eulerr packages. Statistical data evaluation was performed using the ANOVA test in R studio.

### 2.8. Data Visualization and Statistical Analysis

All datasets were analyzed in R, and significance tests were performed with either Wilcoxon or ANOVA with Tukey’s HSD test. The summary details and statistical significance values are listed in individual tables as indicated.

## 3. Results

### 3.1. Developmentally Conserved Spatio-Temporal Replication Pattern in Humans

Replication patterns serve as a visual representation, directly illustrating the spatial organization and temporal sequence of DNA replication [1]. In somatic cells, these patterns have been demonstrated to mirror the level of chromatin organization. Analysis of DNA replication timing profiles (RT profiles) in cell populations, and from individual single cells has unveiled distinctive replication domains spanning 1.5–2.5 megabases (Mb), marked by boundaries between adjacent domains with differing replication timing [3,8,25]. In addition, replication timing provides an excellent platform to study various levels of chromatin organization related to developmental changes [8]. Although RT profiles offer a direct connection to the underlying DNA sequence, they exhibit limitations in temporal resolution, lack 3D spatial information, and do not actually depict the direct relationship between replicating regions and underlying chromatin features. Single-cell microscopy analysis, on the other hand, permits a targeted 4D exploration of DNA replication progression. Crucially, this approach enables the mapping of replication timing for DNA repeat elements, a task challenging for sequencing-based methods, yet these elements constitute a substantial portion of mammalian genomes [6,17,26]. To examine and compare the developmental alterations in the spatio-temporal organization of genome replication in human diploid cells, our initial focus involved analyzing cell cycle progression and S phase duration in human embryonic stem cells (hESC), induced pluripotent stem cells (hiPSC), and somatic cells. To ascertain the doubling time, synonymous with the cell cycle length, we gauged the density of proliferating cells at two distinct time points. Equal numbers of human embryonic stem cells hESC H1, induced pluripotent stem cells hiPSC A4, and hTERT RPE1 cells were seeded, and cell counting commenced every 24 h once the population entered the logarithmic phase of growth. Specifically, for hESC and hiPSC, we waited until a substantial fraction of cells adhered to the surface and initiated colony formation. While the pluripotent cells exhibited a doubling time of around 18 h, the somatic cells showed a doubling time of 24 h (Figure 1B). The durations were comparable to previous reports [27,28]. For determining the S phase duration, we exposed hESC H1, hiPSC A4, and hTERT RPE1 to a 15 min pulse of the nucleoside analog EdU, subsequently detected through click chemistry. EdU facilitated the discrimination between replicating and non-replicating cells. By utilizing DAPI sum intensity as an indicator of total nuclear DNA content and EdU intensity to identify (non)replicating cells, we sorted the cell population into G1, S, or G2 phases (Figure 1A). Using the EdU over DAPI intensity plot we assigned the threshold for EdU. We observed an elevated fraction of replicating cells in pluripotent cell lines compared to the somatic cell line. Through the combination of doubling time and the fraction of the cell population in the S phase, we derived the S phase duration for each cell line by multiplication. Remarkably, despite variations in doubling time and the proportion of replicating cells, the duration of the S phase remained comparable across the different cell lines with a slight increase by 0.5 to 1 h in hTERT RPE1 (Figure 1B). Furthermore, we performed live-cell time-lapse microscopy, to gauge and validate the cell cycle profile and S phase progression by transfecting a plasmid encoding for GFP-tagged PCNA and imaging the transfected cells every 45 min for 24 h (Figure 1E, Videos S1 and S2). The S phase duration was comparable to the data we obtained from cell cycle profiling in Figure 1B.

The spatio-temporal replication patterns in somatic cells have been extensively investigated through live-cell time-lapse microscopy and pulse-chase experiments in fixed cells [1,4]. Notably, beyond the observed similarities in S phase duration, we identified a comparable progression of spatio-temporal replication in both hiPSC A4 and hTERT RPE1 cell lines as shown in Figure 1C. Distinct spatial patterns of replication foci (RFi) were evident in both cell lines as they progressed in S phase. During the early stages of S phase or S I, numerous fine-dotted RFi were dispersed throughout the nucleus, including a few within the nucleolus. This pattern transitioned to the localization of seemingly larger RFi at the nuclear and nucleolar periphery during the middle of S phase or S II, which represented the most extended S phase lasting 4 to 5 h. In the final S phase stage or S III, the RFi increased in size, forming small clusters. A noticeable difference in RFi distribution emerged: in hTERT RPE1, most clustered RFi moved towards the nuclear border from the inner core of the cell, whereas in hESC H1 and hiPSC A4, they moved away from the nuclear border and clustered around and inside the nucleolus. This visually contrasting behavior suggests nuanced differences in the spatio-temporal dynamics of DNA replication between these cell lines.

### 3.2. Characterization of Spatio-Temporal RFi Reveals a Change in Late-Replicating RFi Distribution

To extend our observations in fixed cells, we conducted a pulse-chase–pulse-chase experiment combined with 3D image analysis. Utilizing the known S phase duration, we determined the chase duration after each pulse to encompass most of the S phase. As the total S phase duration was around 11.5 to 12 h, we reasoned that three pulses separated by three hours of chase would cover the main S phase sub-stages. The experimental protocol involved pulsing an asynchronously growing cell population with the nucleoside analog EdU for 15 min, followed by washing to remove excess nucleoside analog. A subsequent chase with thymidine lasted for 3 h. After removing the media, we initiated another pulse with BrdU for 15 min, followed by washing and a subsequent 3 h chase with thymidine before fixing the cells. Replication foci (RFi), marked by EdU, BrdU, and the replisome machinery PCNA, were then detected using click chemistry and (immuno)staining. While EdU and BrdU highlighted the RFi patterns during the respective nucleotide incorporation phases, PCNA marked the active RFi at the time of fixation. This comprehensive approach allowed us to capture the spatial patterns at three distinct time points within individual cells, providing valuable insights into the dynamics of RFi throughout the S phase. Employing confocal microscopy, we captured images of cells at all three timestamps, each corresponding to active replication phases. In this approach, the EdU pattern represented S I, the BrdU pattern represented S II, and the presence of PCNA foci marked S III. This method allowed for the precise visualization of cells undergoing DNA replication across the distinct stages of the cell cycle, providing a detailed spatio-temporal understanding of the replication process (Figure 1C). We used this information to measure the duration of each S phase stage by manually counting the fraction of cells in each S phase and multiplying it by the corresponding doubling time (Figure 1D). We observed a consistent sub-S phase distribution among all cell lines and the S II exhibited a prolonged duration compared to the other stages.

**Figure 2:**
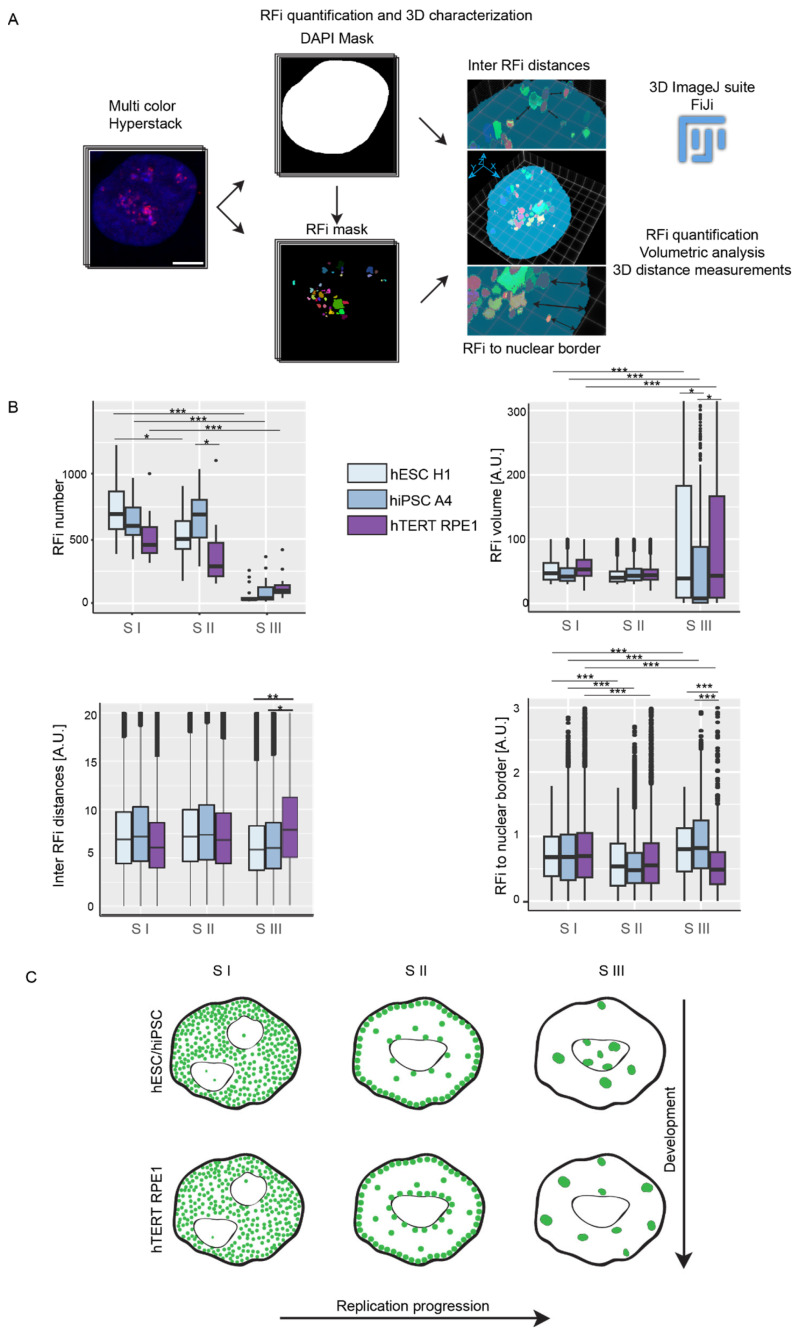
Feature analysis of the replication foci (RFi) in different S phases. (**A**) The illustration shows the image analysis approach for characterizing RFi from different time points in the same cell. Nuclear mask was created using the DNA dye DAPI and applied to the other three channels (EdU, BrdU, and PCNA) before the respective channel segmentation. Within this mask, RFi features were quantified. Scale bar: 5 µm. (**B**) Plots show the number, volume, and distance analysis (inter-RFi and RFi to the nuclear border) of the RFi as the cell progresses through the S phase. (**C**) Illustration shows the subnuclear distribution of RFi and its features in different S phase stages as indicated. The lower and upper whiskers of the boxplot correspond to the 25th and 75th percentiles, the box to the 50th percentile, and the line depicts the median. Statistical significance was performed using ANOVA, and Tukey’s honest significance test (not significant is given for *p*-values ≥ 0.05; one star (*) for *p*-values < 0.05 and ≥ 0.005; two stars (**) is given for values < 0.005 and ≥ 0.0005, and ≥ 0.0005 are given (***); only the significant differences are shown). For more details, see Table S2. Scale bar: 10 µm.

Moving forward, we proceeded to quantify and characterize RFi during different S phases and across various cell lines. We acquired multicolor images in 3D using confocal laser scanning microscope in 100× objective with Z interval of 300 nm (voxel size = 0.125 × 0.125 × 0.29) with enough Z planes to cover the whole nucleus typically between 20–35 stacks. Leveraging the 3D confocal data, we performed segmentation of the RFi, quantified the number of RFi in each S phase, and extracted volumetric and spatial features of the RFi (Figure 2A and Figure S1B). Across all cell lines, the number of RFi was notably higher in S I, exhibited a slight reduction in S II (except hiPSC), and dropped significantly in S III. The volume of the RFi was comparable in S I and S II, but it increased in S III (Figure 2B). Upon comparing the number of RFi among different cell lines, a distinctive pattern emerged. Pluripotent stem cells exhibited a higher number of RFi during both S I and S II phases, with a slight decrease in S III, in comparison to somatic cells. This observation suggests a variable regulation of DNA synthesis in pluripotent stem cells relative to somatic cells, indicative of developmental differences in DNA replication dynamics. Both, pluripotent as well as somatic cells decreased the numbers of RFi in the late S phase S III.

We additionally extracted the 3D inter-RFi distances and the distances from RFi to the nuclear border, which collectively elucidates the 3D spatial patterns (Figure 2A). In pluripotent cells, we observed that inter-RFi distances remained consistent in S I and S II but decreased significantly in S III. Conversely, in pluripotent cells, distances from RFi to the nuclear border decreased from S I to S II and then increased significantly in S III. This observation suggests that in pluripotent stem cells, RFi tend to situate closer to each other, forming a large cluster in the inner core of the nucleus, particularly around and inside the nucleolus, as determined by visual examination. In contrast, in somatic cells, inter-RFi distances increased progressively from S I to S III. Meanwhile, distances from RFi to the nuclear border decreased from S I to S II and dropped even further in S III. This indicates that in somatic cells, the late-replicating RFi, while forming comparable clusters, tend to move away from each other and the nucleolus, ultimately positioning themselves around the nucle(ol)ar border. These distinct spatial dynamics underscore differences in the organization of RFi during different phases of the cell cycle between pluripotent and somatic cells (Figure 2C).

To summarize, while there is not a distinct global switch in the spatio-temporal replication pattern associated with developmental differences, as observed in *Drosophila* or mice, our findings highlight notable differences in the S phase dynamics, particularly in late S phase, between pluripotent stem cells and somatic cells. This analysis contributes to our understanding of underlying DNA replication regulation during development.

### 3.3. Replicon Quantification, Fork Efficiency, and Genome-Wide Origin Mapping Unravel Alterations in the Genome Replication Program across Developmental Transitions

Advanced optical microscopy techniques have revolutionized imaging capabilities, overcoming the resolution limit inherent to traditional light microscopy. Through the application of multicolor 3D structured illumination microscopy (3D-SIM), recent advancements have enabled the resolution of individual replication foci (RFi) down to single replicons. Remarkably, this technique has even allowed, to some extent, the visualization of individual replication forks [6,29].

**Figure 3:**
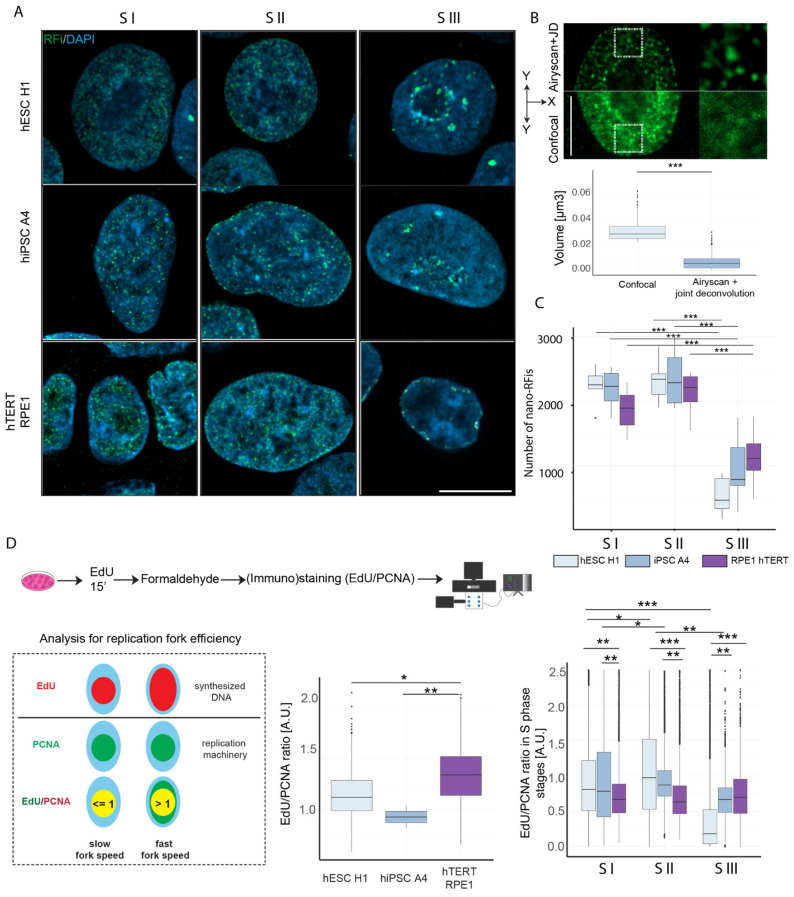
Quantification of the number of replicons and fork speed in S phase stages (**A**) Super-resolution PCNA (green) images overlaid with DAPI (blue) in three S phase stages in hESC H1, hiPSC A4, and hTERT RPE1 are shown. (**B**) Comparison between confocal and super-resolution (ASJD) images of RFi of the same cell and region. The plot shows the comparative volume of (nano)RFi detected in confocal and ASJD mode. (**C**) The plot shows the quantification of the nano-RFi in different S phase stages. (**D**) An illustration depicts the approach to measure the comparative replication fork speed. The EdU was pulsed for 15 min and detected using click chemistry, and PCNA was detected by antibodies. For measuring nucleotide incorporation rate, the ratio of EdU (incorporated nucleotides) and PCNA (active replication) sum intensities was measured as a marker for the speed of replication forks. If the ratio shows a value ≤ 1, this means a complete overlap or localization of EdU inside PCNA and indicates a slow replication fork speed. If the ratio of both signals is > 1, more DNA was synthesized, indicating faster replication fork speed. The middle plot depicts the fork rates of S phase cells across cell lines measured by high-throughput imaging and analysis without discriminating between S phase stages. The right plot shows the fork rate of individual S phase stages measured from high-resolution images across cell lines. The lower and upper whiskers of the boxplot correspond to the 25th and 75th percentiles, the box to the 50th percentile, and the line depicts the median. Statistical significance was performed using ANOVA and Tukey’s honest significance test (not significant is given for *p*-values ≥ 0.05; one star (*) for *p*-values < 0.05 and ≥ 0.005; two stars (**) is given for values < 0.005 and ≥ 0.0005, and 0.0005 to 0 are given (***); only the significant differences are shown). For more details, see Table S3. Scale bar: 10 µm.

We utilized Airyscan SR, in conjunction with Airyscan joint deconvolution (ASJD), to acquire super-resolved multicolor images in 3D (Figure 3A). A comparative analysis of a specific nuclear region with RFi obtained on the same system, first in confocal mode and then in ASJD mode, demonstrates a significant enhancement in resolution, allowing the discernment of nano-scale RFi or replicons (Figure 3B). We observed that the volume of the segmented RFi from confocal images was 4–5 times higher compared to super-resolution imaging (ASJD) (Figure 3B, plot). Hereafter, we refer to the latter as “nano-RFi” and the former as RFi. Through segmentation and quantification, we determined the number of nano-RFi in each S phase and cell line. In general, we observed a 4–5 times increase in the number of nano-RFi compared to confocal microscopy (compare Figure 2B to Figure 3C). During the early S phase, a similar observation of nano-RFi numbers was noted, where pluripotent cells exhibited a higher count of replication origins firing in S I (Figure 3C). In contrast to the confocal data, where RFi numbers were lower in S II compared to S I, we observed a gain in nano-RFi numbers. The total number of active origins was found to be highest in S II across cell lines. Interestingly, nano-RFi numbers were comparable among pluripotent and somatic cells during this phase. In S III, mirroring our earlier observation (Figure 2B), nano-RFi counts were higher in somatic cells than in pluripotent cells.

The attributes of the replication fork, including directionality, speed, and inter-origin distances, are intricately influenced by developmental fate. To gauge fork speed, we employed total signal intensities and conducted a ratiometric analysis of nucleotide incorporation rates per active replisome, as previously described [30]. While PCNA is a component of the DNA replication machinery and is therefore indicative of the number of active replisomes, the quantity of incorporated nucleotides reflects both the number of active replisomes and the replication fork speed. Through the calculation of the ratio between the total nucleotide signal and the total PCNA signal, we assessed variations in the relative replication fork speed between pluripotent and somatic cells. A higher normalized ratio of EdU/PCNA indicates an increased synthesis of DNA per active replisome and, consequently, faster replication forks (Figure 3D). While this approach is not a direct measurement using the classical DNA fiber analysis, and serves as a proxy, it gives us a distinct advantage to assess the fork efficiency in individual cell and S phase sub-stages. The outcome interestingly brings together previously published results using DNA fiber analysis [31]. The latter yielded higher fork speed in somatic cells, which is compatible with our data analyzing the EdU/PCNA ratio by high-throughput imaging taking all S phase cells into consideration (Figure 3D, middle plot). This outcome concomitantly validates this ratiometric approach. Interestingly, using this method we can discriminate S phase sub-stages. We pulsed the hiPSC A4 and hTERT RPE1 with EdU for 15 min and detected EdU and PCNA. Using the aforementioned strategy, we measured the fork speed in S phase sub-stages in all cell types. The outcome of this latter analysis indicated that pluripotent cells (iPSC and ESC) have higher fork speed at the early stages of S phase but decrease the fork speed at later S phase stages (in particular, ESC), whereas somatic cells did not change the fork speed appreciably (Figure 3D, right plot).

**Figure 4:**
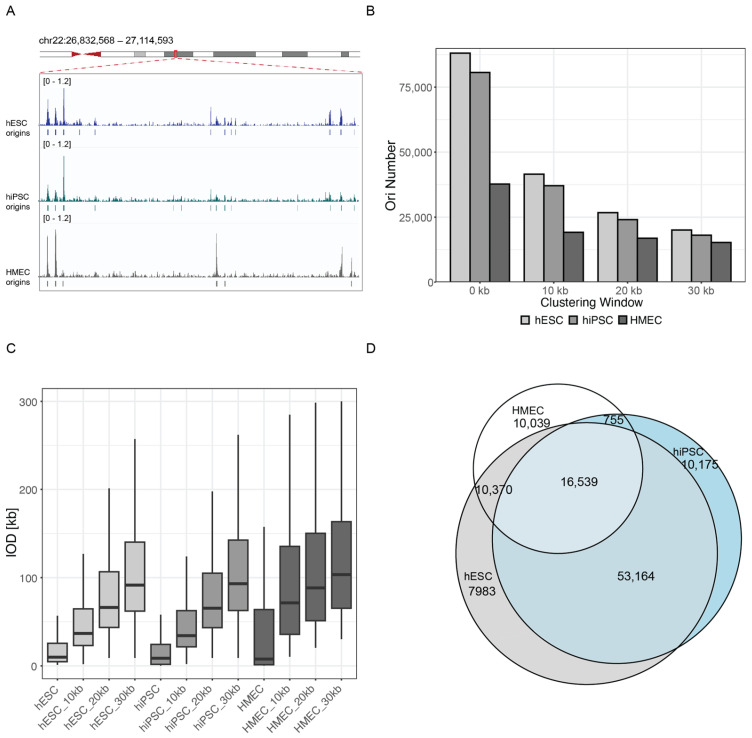
Genome-wide replication origins distribution in selected human cell lines based on the SNS-seq origin mapping method. (**A**) Representative example of replication origins distribution in hESC, hiPSC, and HMEC. The origin profiles correspond to normalized read counts (scale 0–0.7 counts per million). Below the profiles, the origins identified by MACS2 peak callers are shown. (**B**) Comparison of the origin numbers in the human embryonic cell line (hESC), induced pluripotent cells (hiPSC), and the somatic HMEC cell line. Additionally, for the total identified peaks, the graph represents the origin number after clustering of closely situated origins at the distances of 10, 20, and 30 kb. (**C**) Comparison of the inter-origin distances (IOD). The IOD distances were also compared after origin clustering at the distances specified. The statistical evaluation of IODs between different cell lines and same clustering distance were significant (*p*-value < 0.001) with the only exception of the difference between ESC and iPSC, which was not significant *p*-value = 0.4125770. For more details see Table S4. (**D**) Overlap of peaks among the different cell lines.

Next, based on the publicly available short nascent strand isolation and sequencing (SNS-seq) genome-wide datasets originating from two different laboratories (Table 7), we determined the genome-wide position of replication origins in HMEC (human mammary epithelial cells), hESC and hiPSC. The SNS-seq method is a highly resolutive method permitting to map all possible initiation sites at the population level. The replication landscape, including origins localization and origin reads profile in hESC, hiPSC and HMEC in an arbitrary selected genomic region is represented in Figure 4A. Our analysis revealed that hESC and hiPSC cells possess a similar number of potential replication sites, 88,056 and 80633, respectively, whereas HMEC possesses only about half of this number, 37,703 (Figure 4B). HMEC are terminally differentiated mammary epithelial cells in contrast to the pluripotent state of hES and hiPS cells. A similar “disproportion” of origin number at different differentiation stages, has been already observed previously when studying mouse cells [6]. Mouse embryonic stem (mES) cells had approximately the double number of origins as mouse embryonic fibroblasts (MEF). Merging the closely situated origins at 10, 20, and 30 kb (distances in the linear genome), leads to a decrease in the origin number variability between embryonic and somatic cells suggesting that the replication origins tend to be more strictly defined in differentiated cells in comparison to the embryonic cells where the replication initiation seems to be much more flexible within the same genomic region (Figure 4B). Origin clustering at about 30 kb permitted the identification of about 15,000 to 20,000 individual replication origins in all cell types. Next, we analyzed the inter-origin distances (IOD). Consistent with the observed differences in origin numbers, the mean IOD calculated in HMEC was twice as big as the mean found in hESC and hiPSC (Figure 4C). Unclustered origins are situated at a mean distance of 34.3 kb, 37.5 kb, and 79.9 kb, in hESC, hiPSC, and HMEC cells, respectively. This mirrored the earlier studies performed using developmentally different mouse cell lines [6]. Even after clustering of replication origins, IODs in HMEC remained 25% bigger as in hES cells (Figure 4C) (after clustering within 30 kb windows calculated IODs for hESC, hiPSC and HMEC cells were 150.7 kb, 167.5 kb, and 197.5 kb, respectively). Next, we analyzed the number of common origins among the cell lines. We observed a high overlap in the replication origins identified in the different cell lines. hES and iPS cells shared about 86% common origins, 71% of origins identified in HMEC were also present in hES, and about 46% of origins identified in HMEC were also found in hiPSC (Figure 4D).

In conclusion, the human pluripotent stem cells start the replication with a higher number of origins and higher fork speed, which decrease towards the late S phase. The total number of potential initiation sites decreases when cells differentiate and IODs concomitantly tend to increase but replication origins repetitively localize at the same positions.

### 3.4. Chromatin Compaction Analysis and RFi-Associated Histone Modification Measurements Reveal Differential Chromatin Dynamics

**Figure 5:**
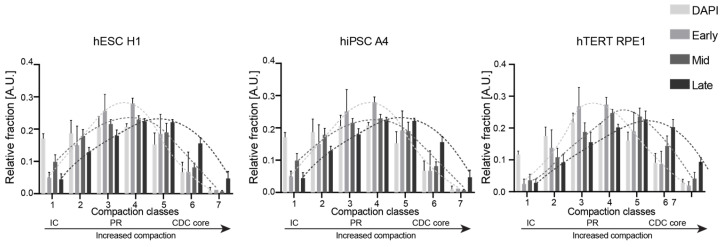
Quantification of chromatin compaction with replication progression across cell lines. RFi in S phase stages were mapped to chromatin compaction classes across cell lines using the statistical tool Nucim on platform R (see Section 2 and Figure S2). Lines connecting data corresponding to the same S phase stages were drawn for easier visualization. Distribution differences on classes *p*-values < 0.005 for all S phase stages for each cell line).

In recent years, various microscopy tools have been employed to dissect chromosome territories (CT) and the fundamental units of CT, known as chromatin domains (CD) [32,33]. The functional nuclear organization is characterized by two spatially co-aligned compartments: the active nuclear compartment (ANC) and the inactive nuclear compartment (INC). The INC consists of compacted portions of chromatin domain clusters (CDCs), or CDC core, which are transcriptionally incompetent and enriched in repressive histone marks. Meanwhile, the ANC encompasses the interchromatin compartment (IC), a largely chromatin-devoid and decondensed region that is transcriptionally competent, serving as the periphery of chromatin domain clusters or the perichromatin region (PR). Using a combined approach of highly resolved microscopy and statistical image analysis, individual nuclei can be subdivided into different DNA compaction classes. We employed the Nucim tool, available on the statistical analysis platform R, which utilizes a hidden Markov random field model to assign each voxel to different compaction classes based on DAPI intensity [24,34]. Notably, this approach is not constrained by staining variations that may arise from different samples and considers individual nuclei (refer to Figure S2A). Subsequently, we segmented and mapped replication foci (RFi) to individual compaction classes using an intensity-weighted threshold method across different S phase stages. In hiPSC A4, we observed a distinct compaction class distribution compared to hTERT RPE1, with a higher fraction of chromatin in the interchromatin compartment (IC) and less in the inactive nuclear compartment (INC), while the active nuclear compartment (ANC) fraction remained comparable (Figure 5). Following the mapping of RFi to compaction classes, hiPSC A4 displayed a similar distribution in S I and S II, with a majority of RFi mapped to IC or PR. In hTERT RPE1, three distinct distribution patterns emerged: S I was more present in classes 2, 3, and 4, (ANC) while S II was enriched in classes 4, 5, and 6 (a fraction in INC). In both cell types, RFi from S III were mapped to INC, represented by classes 5, 6, and 7 (Figure 5).

We extended our analysis to map the correlation of active and inactive chromatin marks, represented by histone post-translational modifications, associated with replication foci (RFi). First, we cataloged the subnuclear distribution of different histone marks from the perspective of developmental changes. Following segmentation, we quantified four histone modifications—H3K9ac, H3K9me3, H3K27me3, and H3K36me3—each indicative of distinct chromatin states. We observed a large fraction of H3K27me3 present in INC in hiPSC A4, and in hTERT RPE1 the enrichment shifted towards ANC. Notably, the transcription elongation mark H3K36me3 was highly enriched in the compacted INC regions along with the constitutive heterochromatin mark H3K9me3 (Figure S4B–D). Next, we mapped the correlation between RFi and histone modifications. The results revealed differential dynamics in histone modifications with S phase progression in the two cell types (Figure S4A,C). Consistently, H3K9ac levels were comparatively higher in S I, while H3K9me3 was more likely associated with RFi in S III. While H3K27me3 levels showed no significant change in hiPSC A4, it was notably associated with a large fraction of RFi in S I in hTERT RPE1, suggesting that a portion of facultative heterochromatin replicates earlier in the S phase. In addition, the distribution of S I in hTERT RPE1 correlates with the distribution of H3K27me3. Notably, H3K36me3, associated with diverse functions such as transcription elongation and forming a bivalent-like chromatin mark distribution alongside H3K9me3, exhibited distinct dynamics [35,36]. In hiPSC A4, a substantial fraction of RFi in both S I and S III showed enrichment in H3K36me3. However, in hTERT RPE1, only the RFi in S III demonstrated a higher association with H3K36me3, suggesting a potential role in heterochromatin dynamics, regulation of replication, and cell fate determination.

### 3.5. Repli-FISH Reveals Developmental Changes in the Replication Timing of Tandem and Interspersed Repeats

In the past two decades, extensive efforts have been devoted to dissecting replication timing, unraveling the principles behind the spatio-temporal regulation of genome replication, chromatin organization, and their association with developmental changes [6,8]. Advances in next-generation sequencing technologies have enabled high-resolution profiling of replication timing, even at the single-cell level [37]. However, a notable limitation of this approach lies in mapping the replication timing of repetitive genomic elements due to the complexity of these sequences. Despite this challenge, recent advancements and the complete human genome sequence have facilitated the mapping of replication timing for certain repetitive elements, such as centromeric sequences [38]. Nevertheless, correlating spatial information with replication timing remains complex. Replication timing of key tandem repeats, particularly rDNA, especially in the context of developmental reprogramming, has remained elusive. To address these challenges, we employed RFi detection coupled with fluorescence in situ hybridization (repli-FISH) as an alternative method to dissect the replication timing, particularly for repetitive genomic elements (Figure S5A,D).

**Figure 6:**
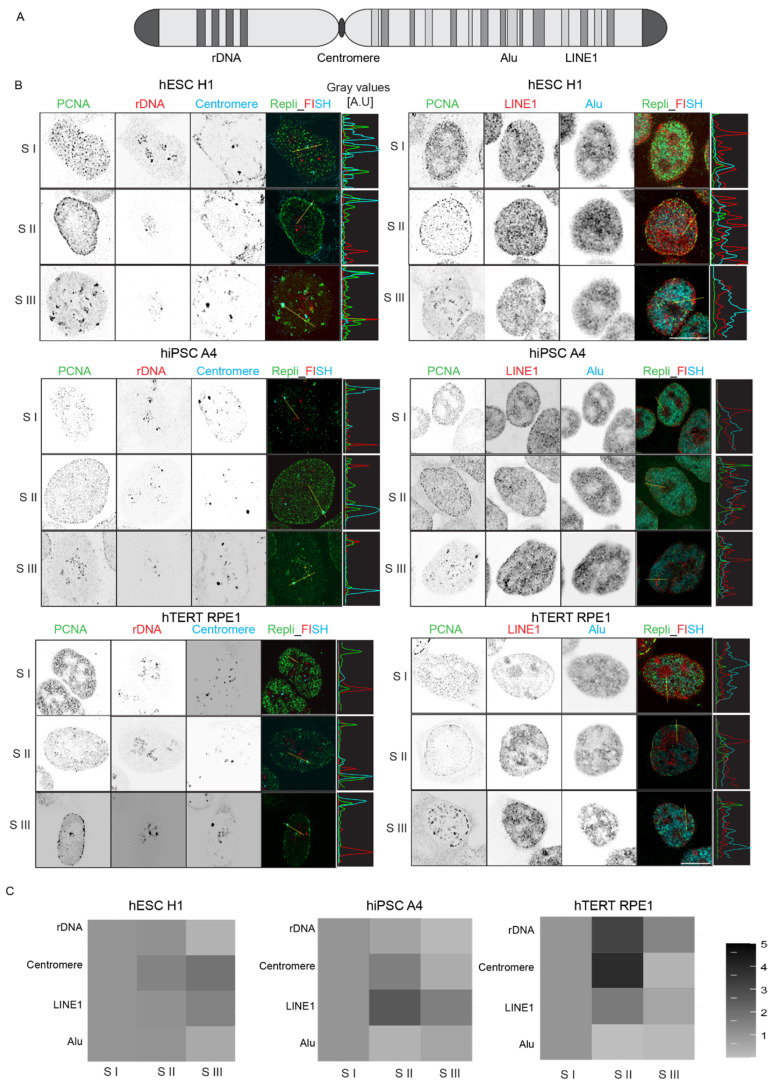
Replication timing of genomic repeat elements. (**A**) Schematic representation of a chromosome with the tandem and interspersed repeat sequences color-coded. (**B**) The co-detection of two combinations of probes across cell lines as indicated (red and cyan) with PCNA (green). The line plots depict the fluorescence intensity distribution of the PCNA and the probes along the line (in microns) drawn on the merged image. (**C**) Heat plot shows the fold change in the sum intensity of each probe replicated in the S phase stages as indicated. The sum intensity of each probe was measured using the segmented RFi as masks in each S phase stage in individual cells and normalized to the median sum intensity of S I for each probe and cell line. For details, see Figure S1B and Table S5. Scale bar: 10 µm.

Using dual FISH coupled with RFi detection through immunostaining for PCNA, we aimed to map the replication timing of interspersed repeats Alu and LINE1, as well as tandem repeats of rDNA and centromere (Figure 6B, Figures S5A–C and S6B). After repli-FISH, we segmented the repeat signals based on the intensity of PCNA in the region of interest (Figure S1B). The relative increase or decrease in intensity represented the replication timing of the corresponding repeats (Figure 6C). Our observations revealed distinct replication dynamics for Alu and LINE1 repeats. Most Alu repeats were replicated in S I in both cell lines, with a fraction also replicating in S III in hiPSC A4. Conversely, most LINE1 elements replicated in S II, but some continued to do so in S III in both cell types. Tandem repeats, specifically centromeres, displayed more defined replication timing in somatic cells, while pluripotent cells exhibited greater plasticity. For example, in both hiPSC A4 and hTERT RPE1, most centromeres replicated in S II, but a small fraction underwent replication in S III. In hiPSC A4, we also observed sporadic RFi colocalizing with centromeres in S I, suggesting that some fraction of centromeres even start replicating earlier in the S phase. These findings of centromere replication in somatic cells align with previously published work [1].

**Figure 7:**
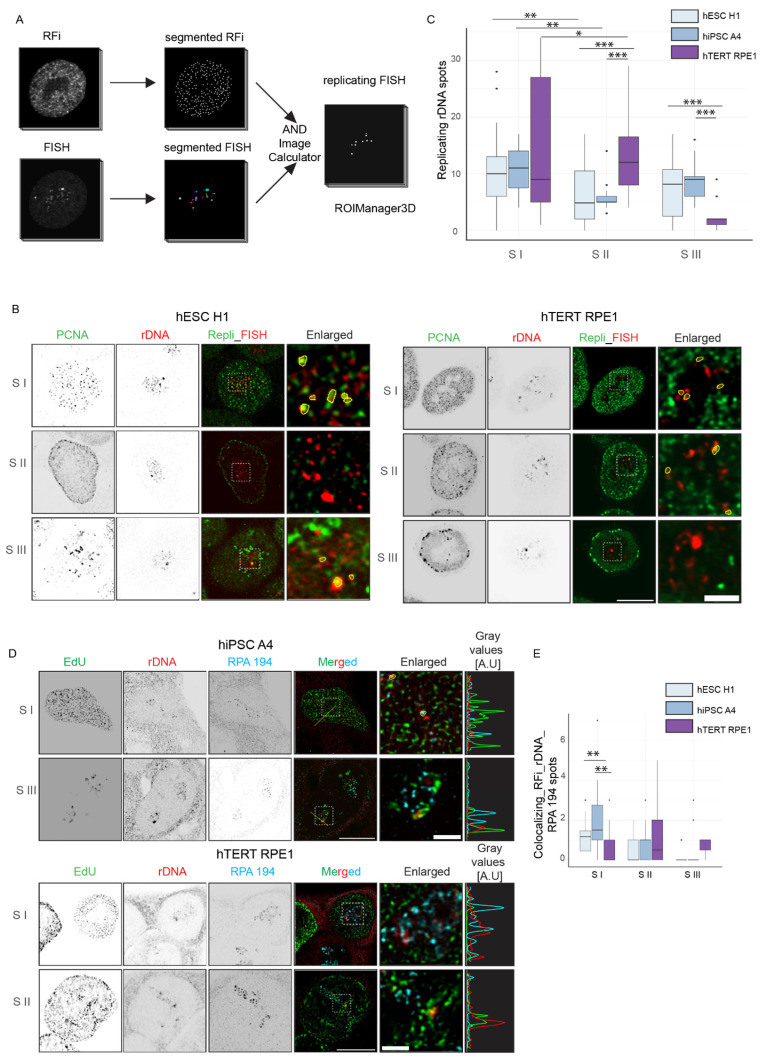
Developmental difference in replication timing of rDNA repeats. (**A**) Analysis pipeline to characterize the rDNA replication timing. RFi and rDNA spots were segmented separately. In FiJi, using the logical function “AND”, both segmented spots were processed to obtain the intersected voxels from both RFi and rDNA, which directly represent the colocalizing rDNA and RFi. (**B**) Images show the PCNA (green), the rDNA (red), and the merged images in different S phase stages as indicated. The contours (yellow) in the enlarged merged image indicate the colocalizing spots. (**C**) The plot depicts the quantification of the replicating rDNA spots in the S phase stages in hPSCs and hTERT RPE1. (**D**) Images show the overlap of RNA polymerase I subunit RPA 194 (representing active transcription) with replicating rDNA repeats in the S phase stages indicated. Contours in the enlarged image show the colocalizing RPA 194 and replicating rDNA as measured with the “AND” logic operation. The line plot shows the intensity distribution of RPA 194 (cyan), rDNA (red), and the EdU (green) along the line. (**E**) The plot shows the number of replicating rDNA spots associated with RPA 194. The lower and upper whiskers of the boxplot correspond to the 25th and 75th percentiles, the box to the 50th percentile, and the line depicts the median. Statistical significance was performed using ANOVA, and Tukey’s honest significance test (not significant is given for *p*-values ≥ 0.05; one star (*) for *p*-values < 0.05 and ≥ 0.005; two stars (**) is given for values < 0.005 and ≥ 0.0005, and 0.0005 to 0 are given (***); only the significant differences are shown). For more details, see Table S6. The scale bar is 10 µm and 2 µm in the enlarged images.

In the case of rDNA replication, a more detailed and nuanced pattern emerged. While in hTERT RPE1, the rDNA replication timing was more defined, and we observed a large fraction replicating in S II, we did not find any specific S phase stage where most of the rDNA replicated in hiPSC A4. When employing the conventional approach of segmenting all probe signals, we encountered limitations, particularly when only a small fraction of repeats were replicating simultaneously. To overcome this, we employed a direct approach, separately segmenting rDNA and RFi signals and performing an “AND” mathematical operation (Figure 7A). This method revealed a switch in rDNA replication timing in hESC H1, hiPSC A4, and hTERT RPE1. While a significant portion of rDNA initiated replication in S I in all cell lines, in hTERT RPE1, the remaining rDNA underwent replication in S II (Figure 6C), consistent with prior findings from mouse models [39]. However, in PSCs, the replication timing of rDNA was delayed, with the remaining repeats replicating in S III and some marking the end of the S phase (Figure 7B,C). We further validated this observation in hESC H1 using repli-FISH coupled with the “AND” analysis approach (Figure S6A). This comprehensive approach provided valuable insights into the dynamics of replication timing for genomic elements that do not replicate in one particular S phase and provided increased temporal resolution.

### 3.6. rDNA Tandem Repeats Show a Switch in Replication Timing and Change in Replication, Transcription Interaction

The rRNA genes are the most transcribed sequences in mammalian cells and are organized in clusters of ribosomal genes called nucleolus organizer regions (NORs) [40]. Super-resolution optical dissection of the rDNA and associated transcription factors like UBF and RNA polymerase I (pol I) subunit RPA 194 shows active tandem repeats organized in a ring-like structure, and these subunits are separated from each other [41]. The pol I, responsible for the rDNA transcription, while more transient in mitotic phases, forms stable clusters organized inside the nucleolus in G1 and through S phase [42]. Single-molecule localization and live-cell confocal microscopy suggest the rDNA transcription and genome replication are separated from each other [43]. Hence, the concomitant rDNA transcription and rDNA replication and their regulatory mechanism draw special attention. We investigated this by co-detecting the rDNA repeats, pol I subunit RPA 194, and active RFi simultaneously (Figure 7D). The rDNA and RPA 194 were generally associated with each other, with the exception of a few rDNA spots, which were comparatively bigger and more condensed. We further analyzed the association of RPA 194 with replicating rDNA spots by separately segmenting rDNA, RPA 194, and RFi and performed “AND” analysis between replicating rDNA obtained as described earlier and RPA 194. This operation resulted in the rDNA spots associated with both transcription and replication (Figure 7E). We generally observed a separation of RPA 194 and RFi in the hTERT RPE1 cell. However, in hiPSC A4, 20–30% of the early-replicating rDNA tended to be associated with the RPA 194, but not the late-replicating rDNA. Overall, the results complement published reports but also highlight differences in terms of the stochastic nature of origin firing in pluripotent cells.

## 4. Conclusions/Discussion

The spatiotemporal regulation of 4D genome replication from the perspective of genome reorganization during developmental processes is poorly understood. Even though past studies have elucidated some fundamental principles of replication programs regarding genes involved in developmental changes, the principles underlying interspersed and tandem repeat element replication are poorly understood. We took advantage of (immuno) repli-FISH and super-resolution microscopy to shed light on the spatio-temporal genome replication progression and investigated in detail the replication dynamics of Alu, LINE1, centromere, and rDNA sequences in developmentally differing cell lines. These data complement the existing understanding of the human developmental regulation of genome replication programs [12,17,44,45].

We first analyzed the spatiotemporal genome replication program using the replication characteristics (S phase duration, number of nano-RFi, IOD, and fork speed) of human pluripotent stem cells (hPSC) and somatic cells. Despite having differential doubling time, the S phase duration was comparable, and a developmentally conserved spatio-temporal pattern was followed where fine-dotted RFi started replicating throughout the nucleus and moved towards the nucleus and nucleolus border while entering into S II. In S III, the RFi increased in size and reduced in number, forming clusters of RFi. The PSC started the S phase with higher origin numbers and fork speed, and both were reduced as the cells entered the late S phase, while the somatic cells decreased the fork speed less drastically throughout the S phase. From the origin mapping analysis, we observed double the number of potential origins and half the inter-origin distance (IOD) size in PSC compared to somatic cells when taking all possible mapped origins (Figure 4).

The availability of complete human genome sequences, development in long-read next-generation sequencing, and powerful algorithms have enabled the mapping of replication timing of human centromeres in individual chromosomes where most of the regions replicate later in the S phase [26,46]. We compared the replication timing of centromeric repeats from repli-FISH with these results and found a similar pattern of S II and S III replicating centromeric repeats. Interestingly, some parts of the centromeric regions also replicated in S I in hPSC. In addition, our results validated earlier replication timing of Alu and LINE1 in somatic cells [17]. We also found a similar result in hPSC but with a higher fraction of LINE1 replicating in S II.

**Figure 8:**
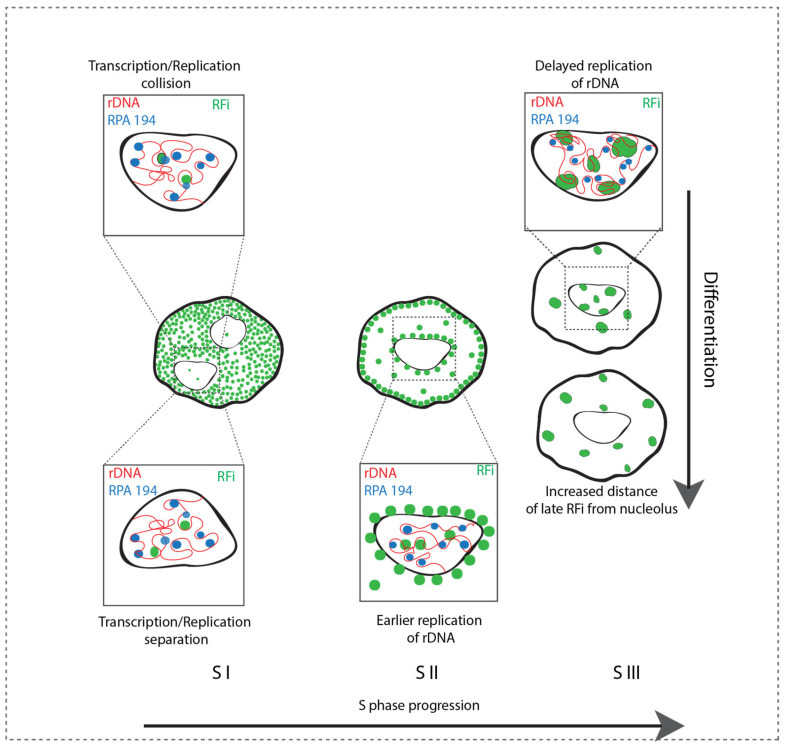
A summary of the developmental difference in genome replication features in pluripotent stem cells (PSC) and somatic cells. The late-replicating RFi cluster around and inside nucleolus in PSC but moves away in somatic cells. The late-replicating rDNA in PSC replicates earlier in S II. The increased origin firing increases the possibility of replication and transcription collision in S I of PSCs. (RFi: replication foci, RPA 194: large subunit of RNA pol I marking active transcription.).

Moreover, we dissected in detail the replication timing of rDNA and its change upon differentiation. While in the *Xenopus* egg system, the rDNA replicates later than the rest of the genome, in mouse embryonic fibroblasts, the rDNA repeats replicate in two waves, with 40% open rDNA replicating early and the rest compacted rDNA later in the S phase [39,47]. We observed a similar trend in human cells. Both somatic and hPSC replicate a fraction of the rDNA repeats in S I. Whereas, in hPSC, the rest of the tandem repeats replicate late in the S III, these replicate earlier in S II in somatic cells. Furthermore, we analyzed the nature of early- or late-replicating rDNA using RPA 194 as a marker for active transcription [41]. We found a general separation between replication and transcription at rDNA repeats in line with earlier observations [43]. However, the early-replicating rDNA tends to be also transcriptionally active, suggesting an increased risk of transcription–replication collision arising by the activation of replication in hPSC. Altogether, our model in Figure 8 complements and expands our understanding of the developmentally regulated genome replication program in human cells.

## Figures and Tables

**Table 1:** List of cell lines.

Name	Species	Type	Gender	Reference
hESC H1	*Homo sapiens*	Embryonic	Male	[13]
hiPSC A4	*Homo sapiens*	iPSC from human neonatal foreskin fibroblast (HFF1)	Male	[14]
hiPSC B4	*Homo sapiens*	iPSC from human neonatal foreskin fibroblast (HFF1)	Male	[14]
hTERT RPE1	*Homo sapiens*	hTERT immortalized retinal pigment epithelial cell	Female	[15]
BJ-5ta	*Homo sapiens*	hTERT immortalized foreskin fibroblasts	Male	[15]

**Table 2:** List of nucleosides and nucleotides.

Name	Application	Detection	Catalog	Company
5-ethynyl-2′-deoxyuridine (EdU)	Labeling of nascent DNA in pulse-chase experiments	ClickIT chemistry	7845.1	Carl Roth, Karlsruhe, Germany
5-bromo-2′-deoxyuridine (BrdU)	Labeling of nascent DNA in pulse-chase experiments	Antibody detection	B5002	Sigma-Aldrich Chemie GmbH, Taufkirchen, Germany
Biotin-16-dUTP	Labeling of FISH probes	Streptavidin	11093070910	Roche Diagnostics Deutschland GmbH, Mannheim, Germany
Cy3-dUTP	Labeling of FISH probes	-	ENZ-42501	Enzo Life Sciences, Lörrach, Germany
Thymidine	Labeling of nascent DNA in pulse-chase experiments, added only in chase period	-	T1895	Sigma-Aldrich Chemie GmbH, Taufkirchen, Germany

**Table 3:** List of antibodies.

Reactivity	Host	Clonality	Dilution	Catalog	Company
Anti-PCNA	Mouse	Monoclonal	1:200	ab29	Abcam, Cambridge, UK
Anti-RPA 194	Mouse	Monoclonal	1:200	sc-48385	Santa Cruz Biotechnology, Dallas, TX, USA
Anti-BrdU	Rabbit	Polyclonal	1:400	600-401-C29	Rockland Immunochemicals, Pottstown, PA, USA
Anti-H3K9me3	Mouse	Monoclonal	1:200	39285	Active Motif, Waterloo, Belgium
Anti-H3K36me3	Rabbit	Polyclonal	1:2000	ab9050	Abcam, Cambridge, UK
Anti-H3K27me3	Mouse	Monoclonal	1:200	61017	Thermo Fisher Scientific, Waltham, MA, USA
Anti-H3K9ac	Rabbit	Polyclonal	1:200	39917	Active Motif, Waterloo, Belgium
Anti-mouse IgG Alexa Fluor 488	Goat	Polyclonal	1:400	A11029	Thermo Fisher Scientific, Waltham, MA, USA
Anti-rabbit IgG Alexa Fluor 488	Goat	Polyclonal	1:500	A-11034	Thermo Fisher Scientific, Waltham, MA, USA
Streptavidin Alexa Fluor 488	Conjugated	-	1:500	S11223	Thermo Fisher Scientific, Waltham, MA, USA
Streptavidin Cy5	Conjugated	-	1:500	PA45001	Amersham Biosciences, Amersham, UK

**Table 4:** List of FISH probes.

Target	Labeling Method	Primers/Plasmids	Reference
Alu	PCR	AluF: 5′-GGATTACAGGYRTGAGCCA-3′ AluR: 3′-RCCAYTGCACTCCAGCCTG-5′	[18]
Centromere	PCR	α27: 5′-CATCACAAAGAAGTTTCTGAGAATGCTTC-3′ α30: 5′-TGCATTCAACTCACAGAGTTGAACCTTCC-3′	[19]
LINE1	Nick translation	Plasmid pLRE3-eGFP	[20]
rDNA	Nick translation	Plasmid pUC-hrDNA-12.0	[21]

**Table 5:** Microscopic systems.

System	Objective	NA	Application	Company
Nikon CREST/TiE2	20x SPlan Fluor LWD DIC (air) or 40X Plan Apo λ DIC (air)	0.7 or 0.95	High-throughput or live-cell time-lapse, wide-field microscopy	Nikon Instruments Inc.,Tokyo, Japan
Leica SP5 II	100X HCX PL APO (oil)	1.44	Confocal laser scanning	Leica GmbH, Mannheim, Germany
LSM 900 Airyscan 2	63x C Plan-Apochromat (oil)	1.4	Confocal and high-resolution	Carl Zeiss AG, Oberkochen, Germany

**Table 6:** List of software.

Name	Version	Platform	Websites	Application	Reference
FiJi	2.14.0/1.54f	MacOS	https://fiji.sc/ (accessed on 25 January 2024)	Image analysis	[22]
StarDist (FiJi)	0.3.0	MacOS	https://github.com/stardist/stardist-imagej (accessed on 25 January 2024)	Nuclei segmentation	[23]
3D suite (FiJi)	1.6	MacOS	https://mcib3d.frama.io/3d-suite-imagej/ (accessed on 25 January 2024)	3D image analysis	[23]
Nucim (R)	1.0.12	MacOS	https://bioimaginggroup.github.io/nucim/ (accessed on 25 January 2024)	Nuclear compaction analysis	[24]
R	4.3.1	MacOS	https://www.r-project.org/ (accessed on 25 January 2024)	Statistical analysis	
Zen	3.9.101	Windows	https://www.zeiss.com/ (accessed on 25 January 2024)	Image acquisition, processing	
Adobe Illustrator 2023	2023	MacOS	https://www.adobe.com/ (accessed on 25 January 2024)	Figure preparation	

**Table 7:** Genome-wide origin mapping.

Dataset	Sample	Characteristics	Method	Cells	Webpage
GSE37757	GSM927236	hESC H9 SNS-seq	SNS-seq	hESC	https://www.ncbi.nlm.nih.gov/geo/query/acc.cgi?acc=GSM927236 (accessed on 25 January 2024)
	GSM927237	hiPSC SNS-seq	SNS-seq	hiPSC	https://www.ncbi.nlm.nih.gov/geo/query/acc.cgi?acc=GSM927237 (accessed on 25 January 2024)
GSE128477	GSM3676411	hESC H9 SNS-seq replicate 1	SNS-seq	hESC	https://www.ncbi.nlm.nih.gov/geo/query/acc.cgi?acc=GSM3676411 (accessed on 25 January 2024)
	GSM3676412	hESC H9 SNS-seq replicate2	SNS-seq	hESC	https://www.ncbi.nlm.nih.gov/geo/query/acc.cgi?acc=GSM3676413 (accessed on 25 January 2024)
	GSM3676413	hESC H9 SNS-seq Control	SNS-seq	hESC	https://www.ncbi.nlm.nih.gov/geo/query/acc.cgi?acc=GSM3676438 (accessed on 25 January 2024)
GSE128477	GSM3676435	HMEC SNS-seq replicate 1	SNS-seq	HMEC	https://www.ncbi.nlm.nih.gov/geo/query/acc.cgi?acc=GSM3676435 (accessed on 25 January 2024)
	GSM3676436	HMEC SNS-seq replicate 2	SNS-seq	HMEC	https://www.ncbi.nlm.nih.gov/geo/query/acc.cgi?acc=GSM3676436 (accessed on 25 January 2024)
	GSM3676437	HMEC SNS-seq replicate 3	SNS-seq	HMEC	https://www.ncbi.nlm.nih.gov/geo/query/acc.cgi?acc=GSM3676437 (accessed on 25 January 2024)
	GSM3676438	HMEC SNS-seq Control	SNS-seq	HMEC	https://www.ncbi.nlm.nih.gov/geo/query/acc.cgi?acc=GSM3676438 (accessed on 25 January 2024)

HMEC—human mammary epithelial cells; hESC—human embryonic stem cells; hiPSC—human induced pluripotent stem cell; SNS-seq—small nascent strand sequencing.

## Data Availability

All the data can be found here: https://tudatalib.ulb.tu-darmstadt.de/handle/tudatalib/4110.2 (accessed on 25 January 2024).

## Supplementary Materials

The following supporting information can be downloaded at: https://www.mdpi.com/article/10.3390/genes15030305/s1, Figure S1: Image analysis pipeline for RFi detection, characterization, and measurements; Figure S2: Image analysis pipeline for mapping RFi to chromatin compaction classes using Nucim package on statistical analysis platform R; Figure S3: S phase progression of hiPSC B4 and BJ-5ta (both diploid); Figure S4: Histone modification dynamics with progressing S phase stages; Figure S5: Metaphase and interphase fluorescence in situ hybridization (FISH) of the repetitive genomic elements; Figure S6: Replication timing of rDNA tandem repeats using conventional and AND methods; Table S1: Statistics parameters of Figure 1; Table S2: Statistics parameters of Figure 2; Table S3: Statistics parameters of Figure 3; Table S4: Statistics parameters of Figure 4; Table S5: Statistics parameters of Figure 6; Table S6: Statistics parameters of Figure 7; Table S7: Statistics parameters of Figure S4; Video S1: Live-cell timelapse of hiPSC A4; Video S2: Live-cell timelapse of hTERT RPE1.
