# Supplementary material for: Developmental Changes in Genome Replication Progression in Pluripotent versus Differentiated Human Cells
Source: Genes (Basel). 2024 Feb 27;15(3):305. doi: 10.3390/genes15030305 (PMC10969796; doi:10.3390/genes15030305)
Supplement: Supplementary file 1 [file genes-15-00305-s001.zip › genes-2868200-supplementary.pdf]

genes

Supplementary Information

Developmental Changes in Genome Replication Progression in Pluripotent versus Differentiated Human Cells

Sunil Kumar Pradhan [1], Teresa Lozoya [1], Paulina Prorok [1], Yue Yuan [2], Anne Lehmkuhl [1], Peng Zhang [2,*] and M. Cristina Cardoso [1,*]

[1] Cell Biology and Epigenetics, Department of Biology, Technical University of Darmstadt, 64287 Darmstadt, Germany; sunil_kumar.pradhan@tu-darmstadt.de (S.K.P.); prorok@bio.tu-darmstadt.de (P.P.)
[2] Center for Tissue Engineering and Stem Cell Research, Guizhou Medical University, Guiyang 550004, China; yuanyue5815@163.com
* Correspondence: peng12zhang@gmc.edu.cn (P.Z.); cardoso@bio.tu-darmstadt.de (M.C.C.)

Citation: Pradhan, S.K.; Lozoya, T.; Prorok, P.; Yuan, Y.; Lehmkuhl, A.; Zhang, P.; Cardoso, M.C. Developmental Changes in Genome Replication Progression in Pluripotent versus Differentiated Human Cells. *Genes* **2024**, *15*, 305. https://doi.org/10.3390/genes15030305

Academic Editor: Heinz-Peter Nasheuer

Received: 26 January 2024
Revised: 22 February 2024
Accepted: 23 February 2024
Published: 27 February 2024

Copyright: © 2024 by the authors. Licensee MDPI, Basel, Switzerland. This article is an open access article distributed under the terms and conditions of the Creative Commons Attribution (CC BY) license (https://creativecommons.org/licenses/by/4.0/).

Abstract: DNA replication is a fundamental process ensuring the maintenance of the genome each time cells divide. This is particularly relevant early in development when cells divide profusely, later giving rise to entire organs. Here, we analyze and compare the genome replication progression in human embryonic stem cells, induced pluripotent stem cells, and differentiated cells. Using single-cell microscopic approaches, we map the spatio-temporal genome replication as a function of chromatin marks/compaction level. Furthermore, we mapped the replication timing of subchromosomal tandem repeat regions and interspersed repeat sequence elements. Albeit the majority of these genomic repeats did not change their replication timing from pluripotent to differentiated cells, we found developmental changes in the replication timing of rDNA repeats. Comparing single-cell super-resolution microscopic data with data from genome-wide sequencing approaches showed comparable numbers of replicons and large overlap in origins numbers and genomic location among developmental states with a generally higher origin variability in pluripotent cells. Using ratiometric analysis of incorporated nucleotides normalized per replisome in single cells, we uncovered differences in fork speed throughout the S phase in pluripotent cells but not in somatic cells. Altogether, our data define similarities and differences on the replication program and characteristics in human cells at different developmental states.

Keywords: human cells; induced pluripotent stem cells; pluripotent embryonic stem cells; genome replication progression; repli-FISH; rDNA; centromere; chromatin compaction

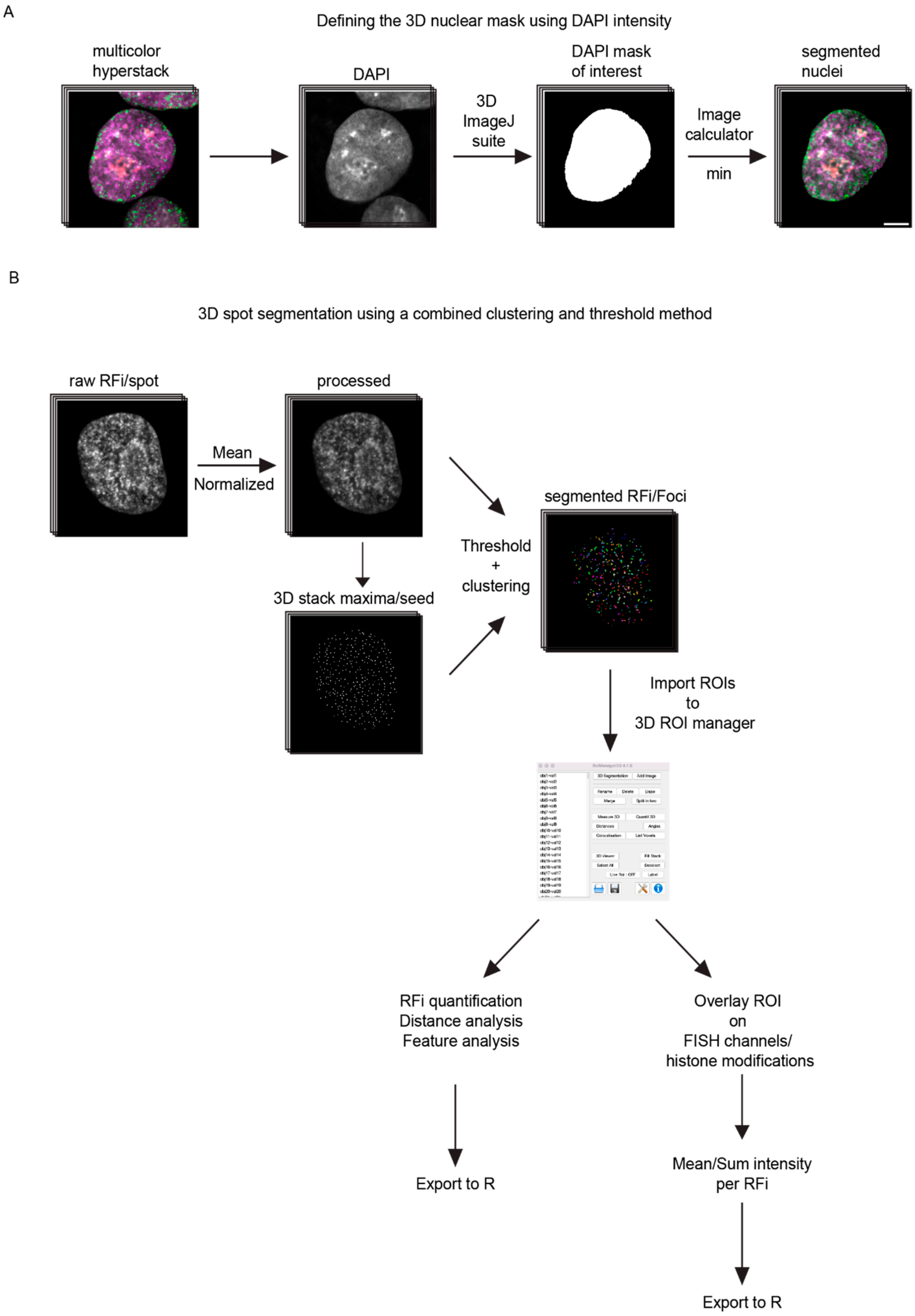

Figure S1. Image analysis pipeline for RFi detection, characterization and measurements. (A) Pre-processing of multicolor hyperstack includes the segmentation of nucleus of interest using DAPI intensity in FiJi (3D ImageJ suite/3D nuclei segmentation). The segmented binary mask defines the ROI using the Image calculator (min) in FiJi. (B) Pipeline explains the process of 3D RFi or spot segmentation. From the processed image, 3D stack maxima points were extracted, the locations were used as seed to cluster. Threshold was applied to filter the seeds before starting to cluster. The segmented RFis were then imported to 3D ROI manager. The number, volumetric, and distance analysis were performed on the 3D ROI manager. The 3D ROIs were overlaid on other channels to get the intensity values of the respective channels.

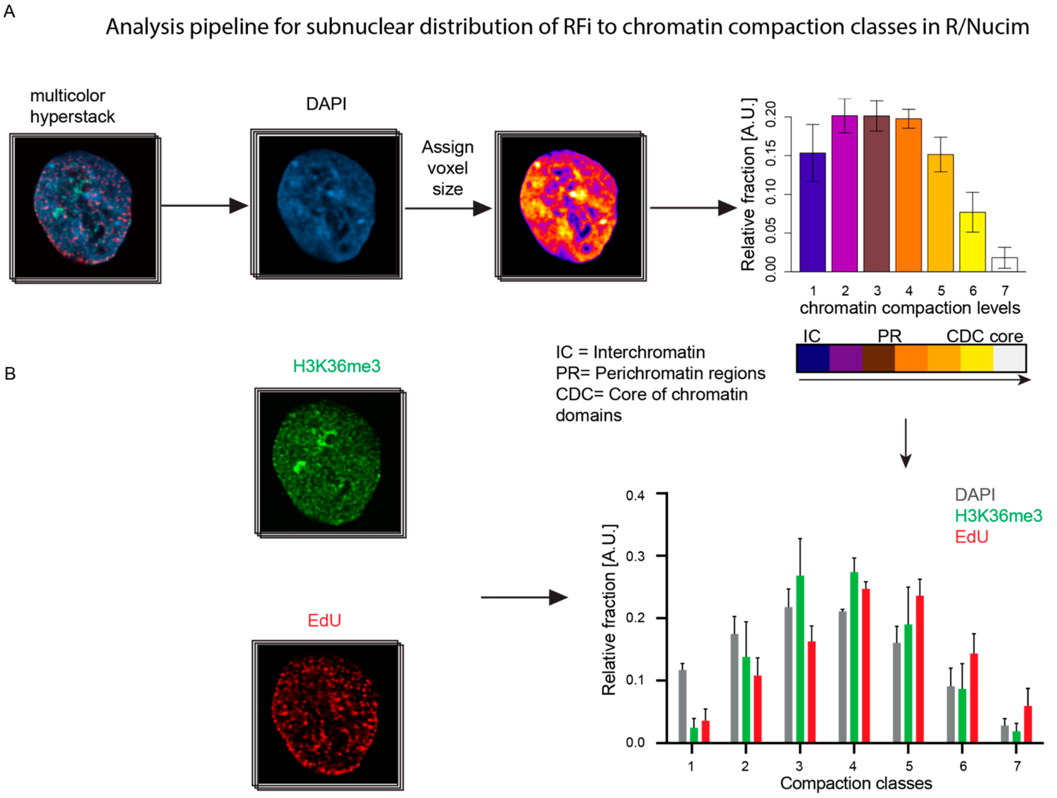

Figure S2. Image analysis pipeline for mapping RFis to chromatin compaction classes using Nucim package on statistical analysis platform R. (A) Pipeline illustrates the import of multicolor hyperstacks to R, using DAPI intensity, to create a nuclear mask, followed by division of individual nucleus into seven chromatin compaction classes (from 1-7, increasing compaction). (B) Using an intensity weighted threshold, the signals from other channels can be mapped to individual compaction classes and relative fraction can be measured. For details see the methods.

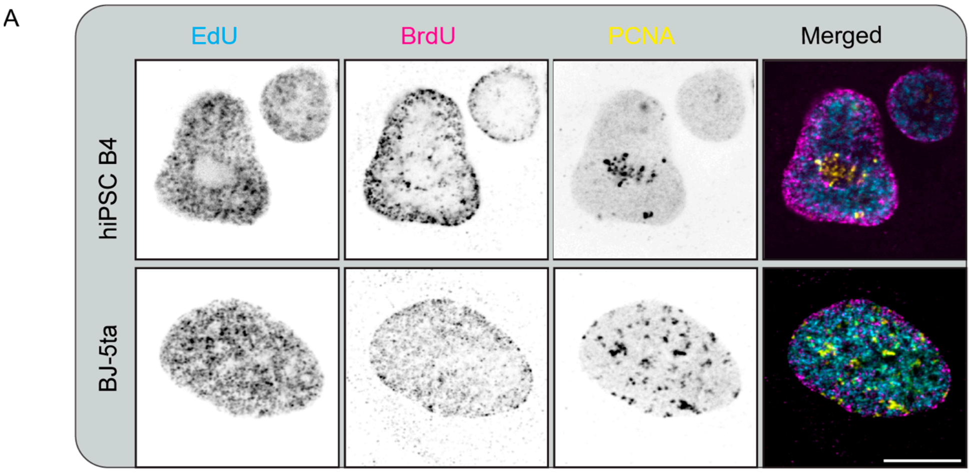

Figure S3. S phase progression of hiPSC B4 and BJ-5ta (both diploid). (A) A pulse-chase pulse-chase experiment, followed by detection of nucleotide analogs and PCNA, and images using confocal microscopy shows the spatial pattern of RFi in three-time points.

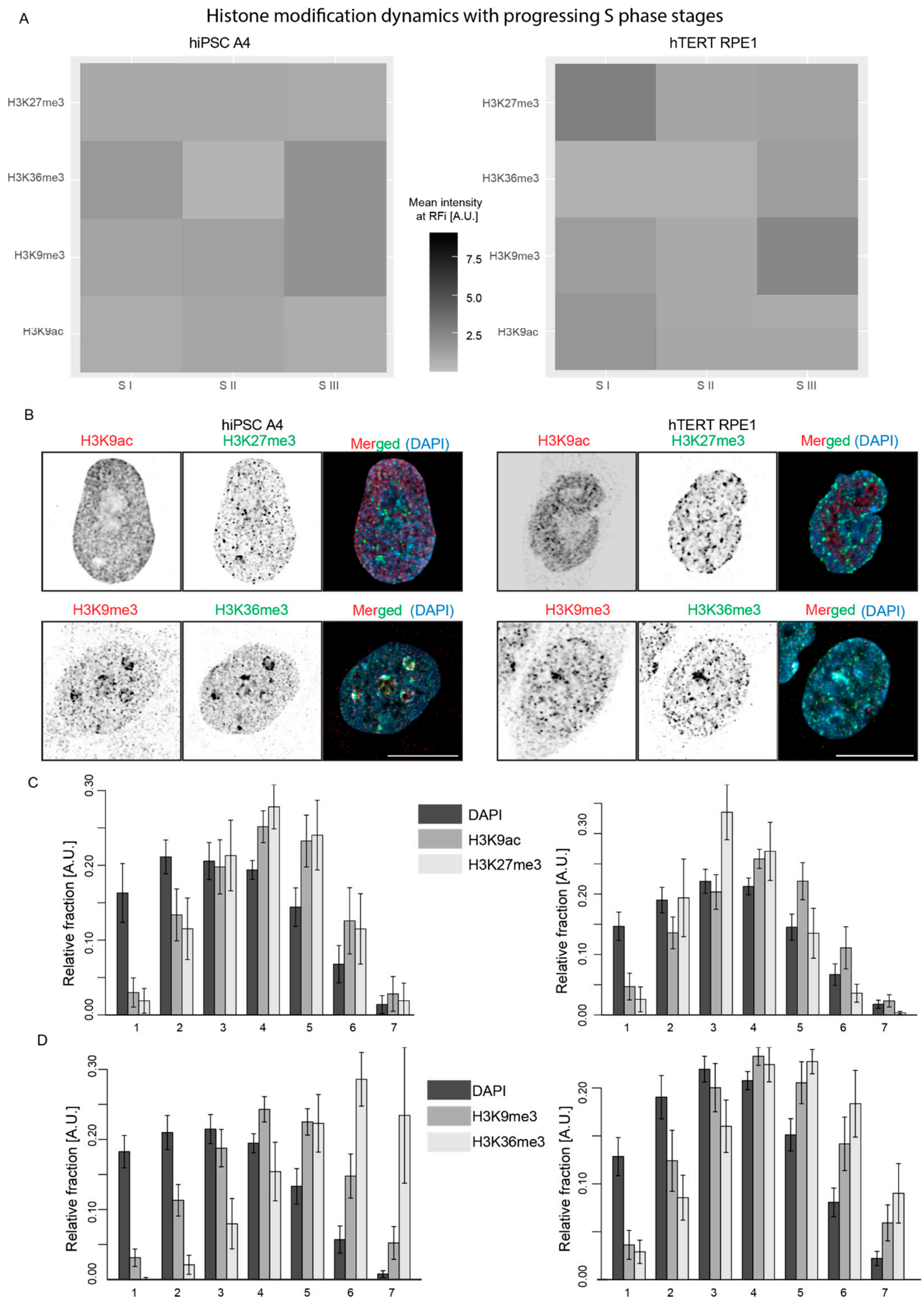

Figure S4. Histone modification dynamics with progressing S phase stages. (A) Heatplot depicts the fold change of the sum intensity of histone modification enriched in individual S phase stages as indicated. The mean intensity of each histone modification was measured using the segmented RFi as masks in S phase stages in individual cells and normalized per histone modification and cell line. For details, see Figure S1 B. (B) Representative images of histone modifications in hiPSC A4, and hTERT RPE1 as indicated. (C) Relative fraction of the euchromatin mark H3K9ac, and the facultative heterochromatin mark H3K27me3 to compaction classes. (D) Relative fraction of the heterochromatin marks H3K9me3, and H3K36me3 in individual compaction classes. For detail regarding mapping see Figure S2. Scale bar: 10 µm.

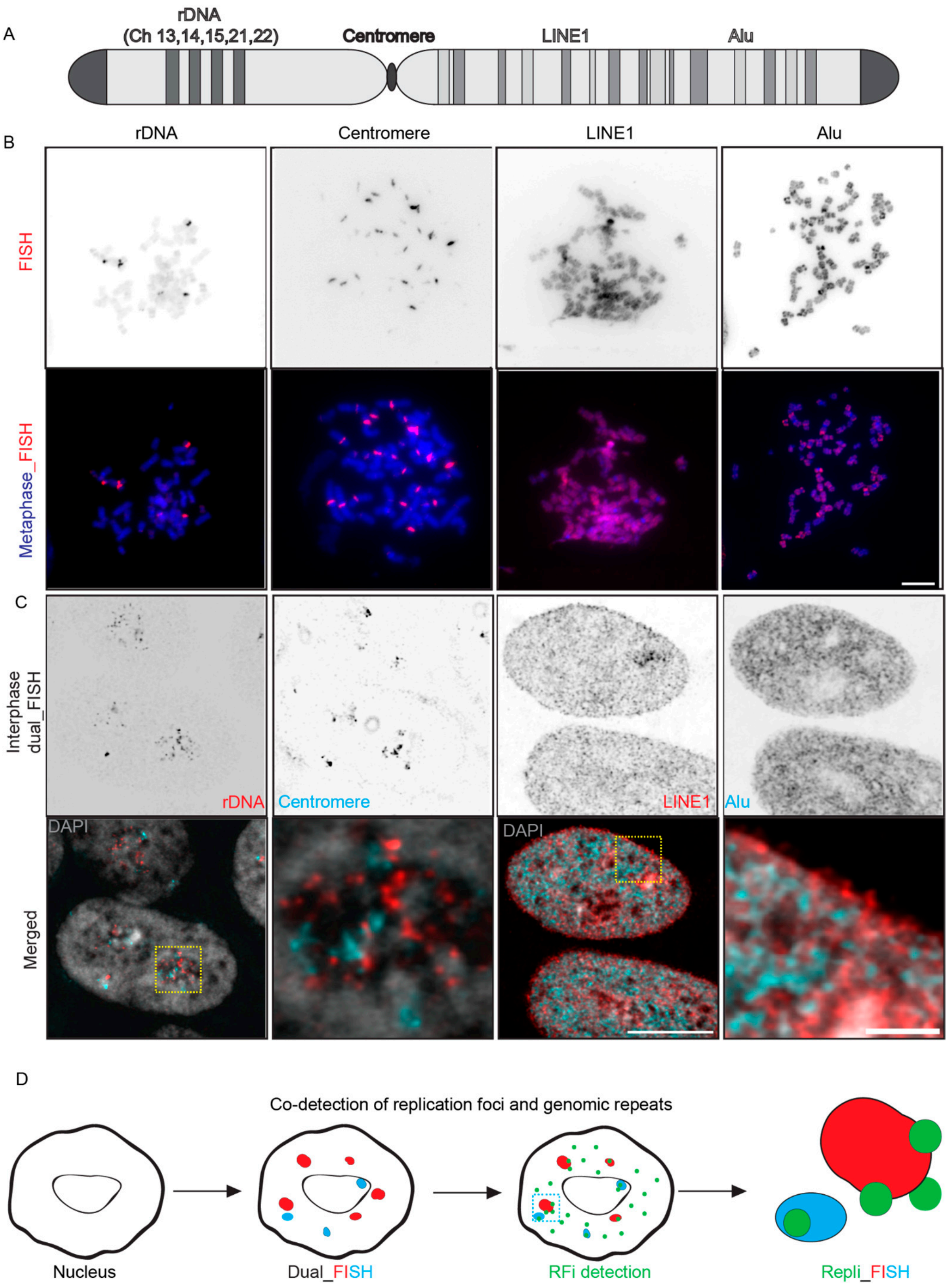

Figure S5. Metaphase and interphase fluorescence in situ hybridization (FISH) of the repetitive genomic elements. (A) Illustration of a chromosome shows the localization of tandem and interspersed genomic repeats. (B) Images show the FISH of rDNA, centromere, LINE1, and Alu on metaphase spread from hTERT RPE1. (C) Example images, and enlarged images show the dual FISH performed on the interphase nucleus. Scale bar is 10 µm in large, and 2 µm in enlarged images. (D) The illustration depicts the Repli-FISH co-detection of active replication foci/RFi and DNA probes using fluorescence in situ hybridization analysis to characterize the replication timing of the targeted genomic repeats.

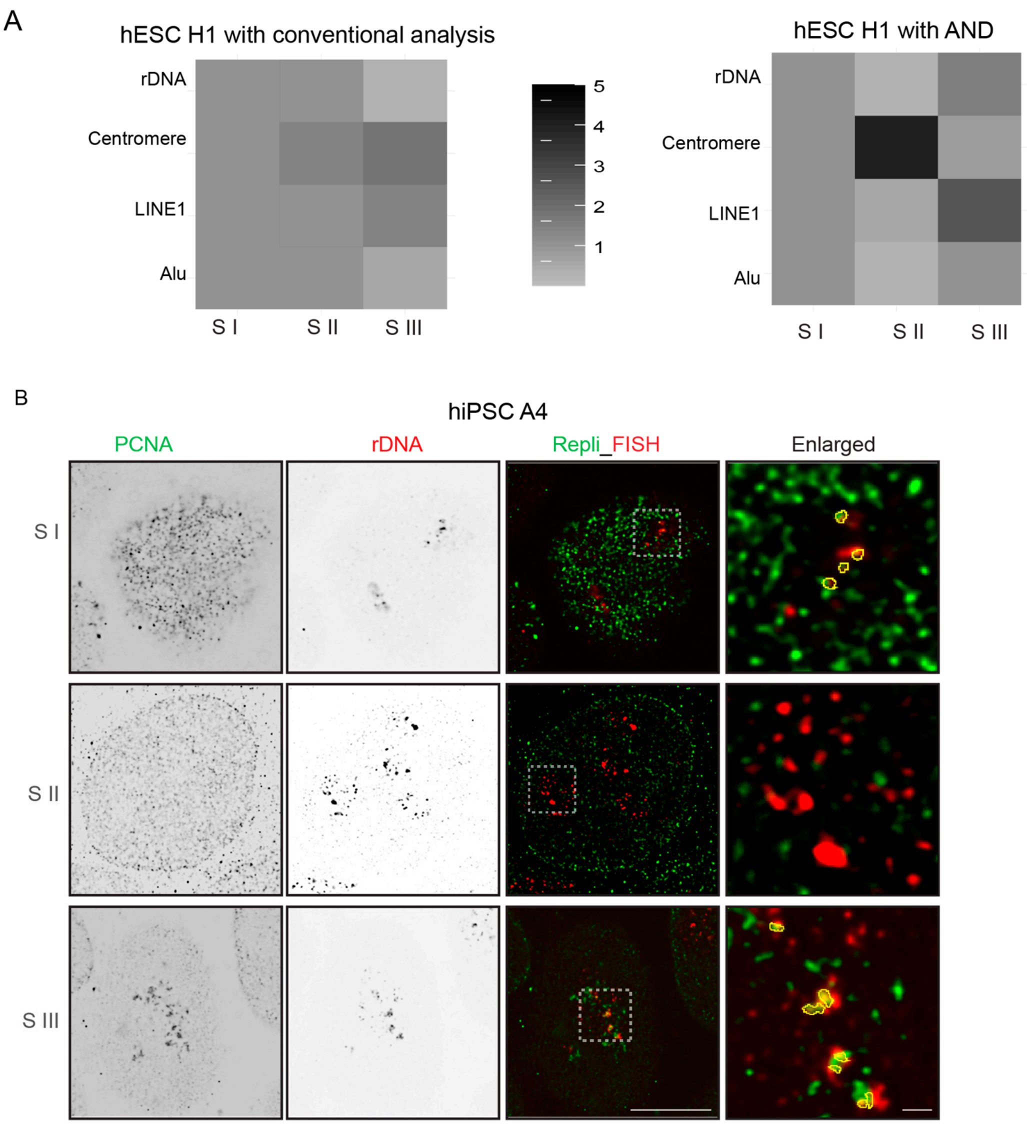

Figure S6. Repli-FISH analysis of interspersed and tandem repeats using conventional and AND methods. (A) Heat plots show the comparison between the conventional method by RFi intensity measurement at the segmented probe. The AND method uses a separately segmented probe, and RFi followed by colocalizing object quantification (B) The images show the representative images of repli-FISH between rDNA repeats and PCNA (RFi) in hiPSC A4. The contours in the enlarged images depict the replicating rDNA in respective S phase stages.

Table S1. Statistics parameters of Figure 1.

Figure	Cell type	Cell cycle	N	EdU Mean	EdU Median	SD
Cell cycle profiling	hESC H1	S	1394	5331.50	5179.49	1138.77
	hiPSC A4	S	1132	3988.85	3988.85	309.34
	hTERT RPE1	S	2409	6336.59	6154.67	1726.99
	hESC H1	G1/G2	1209	2108.154	2027.53	584.14
	hiPSC A4	G1/G2	1048	1701.61	1553.78	615.68
	hTERT RPE1	G1/G2	2585	2979.96	3035.81	355.93

Table S2. Statistics parameters of Figure 2.

Figure	Cell type	S phase stages	N	Mean	Median	SD	*p*-value
RFi number (2 B)	hESC H1	S I	20	729	694	222	vs hiPCS A4 0.91 vs hTERT RPE1 0.81
	hiPSC A4	S I	12	636	603	195	vs hTERT RPE1 0.03
	hTERT RPE1	S I	15	522	456	182	-
	hESC H1	S II	20	502	501	192	vs hiPCS A4 0.27 vs hTERT RPE1 0.0
	hiPSC A4	S II	12	669	691	241	vs hTERT RPE1 0.5
	hTERT RPE1	S II	15	374	288	247	-
	hESC H1	S III	20	59.40	32.50	67.90	vs hiPCS A4 1.0 vs hTERT RPE1 1.0

	hiPSC A4	S III	12	101	41	121	vs hTERT RPE1 0.96
	hTERT RPE1	S III	15	131	99	96.20	-
	hESC H1	S I	20	52	47	18	vs hiPCS A4 1.0 vs hTERT RPE1 1.0
	hiPSC A4	S I	12	47.30	42	16.40	vs hTERT RPE1 1.0
	hTERT RPE1	S I	15	55.40	53	18.50	-
RFi volume (2 B)	hESC H1	S II	20	44.30	40	13.30	vs hiPCS A4 1.0 vs hTERT RPE1 1.0
	hiPSC A4	S II	12	47	43	14.60	vs hTERT RPE1 1.0
	hTERT RPE1	S II	15	46.80	44	13.60	-
	hESC H1	S III	20	397	39	2390	vs hiPCS A4 0.09 vs hTERT RPE1 0.0

	hiPSC A4	S III	12	571	8	10015	vs hTERT RPE1 0.14
	hTERT RPE1	S III	15	252	43	689	-
Inter RFi distances (2 B)	hESC H1	S I	20	7.29	6.90	3.68	vs hiPCS A4 0.0 vs hTERT RPE1 0.0
	hiPSC A4	S I	12	7.88	7.22	4.18	vs hTERT RPE1 0.0
	hTERT RPE1	S I	15	11.60	6.25	25.90	-
	hESC H1	S II	20	7.54	7.18	3.75	vs hiPCS A4 0.0 vs hTERT RPE1 0.0
	hiPSC A4	S II	12	8.02	7.42	4.17	vs hTERT RPE1 0.0
	hTERT RPE1	S II	15	17.50	7.29	38.60	-
	hESC H1	S III	20	6.35	5.84	3.460	vs hiPCS A4 0.0 vs hTERT RPE1 0.0

	hiPSC A4	S III	12	6.76	6.03	3.87	vs hTERT RPE1 0.0
	hTERT RPE1	S III	15	10.80	8.04	19.10	-
RFi to nuclear border (2 B)	hESC H1	S I	20	0.70	0.68	0.40	vs hiPCS A4 0.0 vs hTERT RPE1 0.0
	hiPSC A4	S I	12	0.73	0.68	0.49	vs hTERT RPE1 0.0
	hTERT RPE1	S I	15	0.89	0.72	0.79	-
	hESC H1	S II	20	0.60	0.54	0.39	vs hiPCS A4 0.13 vs hTERT RPE1 0.0
	hiPSC A4	S II	12	0.57	0.48	0.40	vs hTERT RPE1 0.0
	hTERT RPE1	S II	15	0.78	0.58	0.79	-
	hESC H1	S III	20	0.80	0.80	0.42	vs hiPCS A4 0.0 vs hTERT RPE1 0.0

hiPSC A4	S III	12	0.93	0.82	0.60	vs hTERT RPE1 0.0
hTERT RPE1	S III	15	0.60	0.50	0.59	-

Table S3. Statistics parameters of Figure 3.

Figure	Cell type	S phase stages	N	Mean	Median	SD	*p*-value
Number of nano-RFis (3 C)	hESC H1	S I	10	2194	2232	312	vs hiPCS A4 1.0 vs hTERT RPE1 0.81
	hiPSC A4	S I	10	1939	1934	291	vs hTERT RPE1 0.86
	hTERT RPE1	S I	10	1866	1894	341	-
	hESC H1	S II	10	2350	2228	465	vs hiPCS A4 1.0 vs hTERT RPE1 0.99
	hiPSC A4	S II	10	2063	2040	347	vs hTERT RPE1 0.98
	hTERT RPE1	S II	10	2138	2259	345	-
	hESC H1	S III	10	996	787	476	vs hiPCS A4 0.43 vs hTERT RPE1 0.16
	hiPSC A4	S III	10	936	983	412	vs hTERT RPE1 0.99
	hTERT RPE1	S III	10	1111	1112	387	-
EdU/PCNA High-throughput (3 D)	hESC H1	S	1394	1.35	1.02	2.08	vs hiPCS A4 1.0 vs hTERT RPE1 0.04

	hiPSC A4	S	1132	1.41	0.92	1.86	vs hTERT RPE1 0.01
	hTERT RPE1	S	2409	1.75	1.54	0.47	-
EdU/PCNA ratio (3 D)	hESC H1	S I	12	1.19	0.87	1.15	vs hiPCS A4 0.99 vs hTERT RPE1 0.01
	hiPSC A4	S I	16	3.85	1.14	1.50	vs hTERT RPE1 0.0
	hTERT RPE1	S I	10	0.76	0.68	0.49	-
	hESC H1	S II	16	2.17	1.33	2.59	vs hiPCS A4 0.99 vs hTERT RPE1 0.0
	hiPSC A4	S II	10	0.99	0.88	0.49	vs hTERT RPE1 0.52
	hTERT RPE1	S II	10	0.75	0.64	0.46	-
	hESC H1	S III	11	0.96	0.25	1.75	vs hiPCS A4 0.01 vs hTERT RPE1 0.0
	hiPSC A4	S III	23	0.71	0.73	0.32	vs hTERT RPE1 1.0
	hTERT RPE1	S III	14	0.74	0.70	0.39	-

Table S4. Statistics parameters for Figure 4.

**Figure**	**Sample**	**Number of oris**	**Median IOD [kb]**	**Mean [kb]**	**SD [kb]**	***p*-value**
Origin number, inter-origin	hESC 0 kb	88056	10.04	33.43	164.0	vs iPCS 0.41 vs HMEC 0.0
	hiPSC 0 kb	80633	8.94	37.46	185.8	vs HMEC 0.0
	HMEC 0 kb	37703	11.76	79.92	281.0	-
	hESC 10 kb	41523	37.87	72.81	234.1	vs iPC_10kb 0.0

distances (4B, C)						vs HMEC_10kb 0.0
	hiPSC 10 kb	37081	35.73	81.49	268.	vs HMEC_10kb 0.0
	HMEC 10 kb	19165	86.09	157.32	379.1	-
	hESC 20 kb	26718	69.23	113.18	289.6	vs iPC_20kb 0.0 vs HMEC_20 kb 0.0
	hiPSC 20 kb	24043	69.66	125.71	327.6	vs HMEC_20 kb 0.0
	HMEC 20 kb	16901	105.01	178.42	399.8	-
	hESC 30 kb	20035	98.39	150.72	335.2	vs iPC_30kb 0.0 vs HMEC_30 kb 0.0
	hiPSC 30 kb	18042	101.45	167.46	375.3	vs HMEC_30 kb 0.0
	HMEC 30 kb	15265	123.41	197.55	417.4	-

HMEC- human mammary epithelial cells.

Table S5. Statistics parameters of Figure 6.

Figure	Probe	Cell type	S phase stages	N	Mean	Median	SD
Repli-FISH heatplot (5 D)	Alu	hESC H1	S I	19	59.42	54.59	28.18
			S II	18	57.92	52.75	29.64
			S III	22	62.25	57.99	29.19
		hiPSC A4	S I	18	84.91	85.93	23.66
			S II	15	68.48	68.56	19.65
			S III	15	64.91	68.55	24.99
		hTERT RPE1	S I	11	62.09	62.26	13
			S II	14	46.44	44.87	10.07
			S III	11	42.92	43.94	8.47
		hESC H1	S I	19	36.11	32.39	18.36

	LINE1		S II	18	39.13	35.25	20.01
			S III	22	40.59	37.30	19.44
		hiPSC A4	S I	11	37.90	36.30	14.48
			S II	17	41.72	39.98	15.58
			S III	16	32.54	31.52	9.56
		hTERT RPE1	S I	17	58.79	59.82	18.27
			S II	14	65.60	61.70	28.30
			S III	16	49.14	51.67	11.46
	Centromere	hESC H1	S I	35	1.33	0.31	4.36
			S II	27	1.54	0.54	4.48
			S III	8	1.73	0.55	6.70
		hiPSC A4	S I	13	50.12	51.86	22.97
			S II	11	53.14	51.47	15.92
			S III	18	36.95	30.94	22.55
		hTERT RPE1	S I	10	41.54	39.57	17.68
			S II	13	62.66	57.11	27.33
			S III	13	52.81	49.03	23.22
	rDNA	hESC H1	S I	35	5.19	3.27	8.38
			S II	27	4.87	3.48	5.92
			S III	8	5.06	3.42	7.39
		hiPSC A4	S I	15	46.55	44	17.07
			S II	10	43.34	38.69	15.73
			S III	13	46.60	40.78	26.54
		hTERT RPE1	S I	16	50.76	46.8	19.59
			S II	12	37.28	30.15	22.90
			S III	13	37.30	31	22.91

Table S6. Statistics parameters of Figure 7.

**Figure**	**Cell type**	**S phase stages**	**N**	**Mean**	**Median**	**SD**	***p*-value**
Replicating rDNA spots (6 C)	hESC H1	S I	35	10.32	10	6.08	vs hiPSC A4 1.0 vs hTERT RPE1 0.66
	hiPSC A4	S I	19	10.68	11	4.15	vs hTERT RPE1 0.86

	hTERT RPE1	S I	13	13.92	9	12.25	-
	hESC H1	S II	27	6.81	6	5.66	vs hiPSC A4 0.99 vs hTERT RPE1 0.03
	hiPSC A4	S II	13	5.85	5	2.82	vs hTERT RPE1 0.05
	hTERT RPE1	S II	20	12.70	12	5.97	-
	hESC H1	S III	8	7.25	7	5.95	vs hiPSC A4 0.99 vs hTERT RPE1 0.83
	hiPSC A4	S III	12	8.75	9	3.57	vs hTERT RPE1 0.37
	hTERT RPE1	S III	9	2.67	2	2.96	-
Colocalizing rDNA & RPA 194 spots (6 E)	hESC H1	S I	23	1.65	1.87	0.98	vs hiPSC A4 0.0 vs hTERT RPE1 1.0
	hiPSC A4	S I	14	2.14	1.5	1.83	vs hTERT RPE1 0.02
	hTERT RPE1	S I	11	0.64	0	1.03	-

	hESC H1	S II	12	0.42	0	0.69	vs hiPSC A4 0.99 vs hTERT RPE1 0.75
	hiPSC A4	S II	13	0.85	1	0.99	vs hTERT RPE1 0.99
	hTERT RPE1	S II	12	1.17	0.5	1.59	-
	hESC H1	S III	22	0.23	0	0.43	vs hiPSC A4 1.0 vs hTERT RPE1 0.99
	hiPSC A4	S III	14	0.36	0	0.93	vs hTERT RPE1 1.00
	hTERT RPE1	S III	3	0.67	1	0.58	-

Table S7. Statistics parameters of Figure S4.

Figure	Histone mark	Cell type	S phase stages	N	Mean	Median	SD
Histone modification heatplot (S. 3 A)	H3K9ac	hiPSC A4	S I	14	89.03	87.39	29.63
			S II	10	77.77	84.93	40.71
			S III	20	80.03	78.45	27.77
		hTERT RPE1	S I	9	46.18	42.41	23.83
			S II	10	41.27	39.10	17.22
			S III	6	29.06	27	13.28
	H3K9me3	hiPSC A4	S I	14	14.99	12.30	10.95
			S II	8	14.53	11.43	11.25
			S III	14	16.46	9.88	17.24
		hTERT RPE1	S I	10	27.29	25.11	14.66
			S II	5	21.24	18.52	11.08
			S III	15	24.81	22.36	13.41

H3K36me3	hiPSC A4	S I	14	24.83	22.06	12.91
		S II	8	24.54	21.66	13.73
		S III	14	29.42	24.72	18.50
	hTERT RPE1	S I	10	16.23	13.44	11.37
		S II	5	12.84	9.87	10.57
		S III	15	12.76	10.24	10.06
H3K27me3	hiPSC A4	S I	14	23.52	21.85	11.10
		S II	10	19.21	17.58	9.68
		S III	20	14.40	13.28	7.27
	hTERT RPE1	S I	9	24.99	20.91	15.57
		S II	10	24.23	19.75	17.03
		S III	6	24.18	18.42	18.89
